# *ITGB5* and *AGFG1* variants are associated with severity of airway responsiveness

**DOI:** 10.1186/1471-2350-14-86

**Published:** 2013-08-28

**Authors:** Blanca E Himes, Weiliang Qiu, Barbara Klanderman, John Ziniti, Jody Senter-Sylvia, Stanley J Szefler, Robert F Lemanske, Jr, Robert S Zeiger, Robert C Strunk, Fernando D Martinez, Homer Boushey, Vernon M Chinchilli, Elliot Israel, David Mauger, Gerard H Koppelman, Maartje AE Nieuwenhuis, Dirkje S Postma, Judith M Vonk, Nicholas Rafaels, Nadia N Hansel, Kathleen Barnes, Benjamin Raby, Kelan G Tantisira, Scott T Weiss

**Affiliations:** 1 Channing Division of Network Medicine, Brigham and Women’s Hospital and Harvard Medical School, Boston, MA, USA; 2 Partners HealthCare Center for Personalized Genetic Medicine and Harvard Medical School, Boston, MA, USA; 3 Children’s Hospital Informatics Program, Boston, MA, USA; 4 National Jewish Health and University of Colorado Denver School of Medicine, Denver, CO, USA; 5 University of Wisconsin, Clinical Science Center, Madison, WI, USA; 6 Kaiser Permanente Southern California Region, San Diego, CA, USA; 7 Washington University School of Medicine, St. Louis, MO, USA; 8 Arizona Respiratory Center, University of Arizona, College of Medicine, Tucson, AZ, USA; 9 Division of Pulmonary/Critical Care and Allergy/Immunology, Department of Medicine, University of California at San Francisco, San Francisco, CA, USA; 10 Department of Biostatistics, Penn State College of Medicine, Hershey, PA, USA; 11 Division of Pulmonary and Critical Care Medicine, Department of Medicine, Brigham and Women’s Hospital, Boston, MA, USA; 12 Department of Pediatric Pulmonology and Pediatric Allergology, Beatrix Children’s Hospital, GRIAC Research Institute, University of Groningen, University Medical Center Groningen, Groningen, the Netherlands; 13 Department of Pulmonology and Tuberculosis, GRIAC Research Institute, University of Groningen, University Medical Center Groningen, Groningen, the Netherlands; 14 Department of Epidemiology, GRIAC Research Institute, University of Groningen, University Medical Center Groningen, Groningen, the Netherlands; 15 Department of Medicine, Johns Hopkins University, Baltimore, MD, USA

**Keywords:** Asthma, Airway hyperresponsiveness, Genome-wide association study, *ITGB5*, *AGFG1*

## Abstract

**Background:**

Airway hyperresponsiveness (AHR), a primary characteristic of asthma, involves increased airway smooth muscle contractility in response to certain exposures. We sought to determine whether common genetic variants were associated with AHR severity.

**Methods:**

A genome-wide association study (GWAS) of AHR, quantified as the natural log of the dosage of methacholine causing a 20% drop in FEV_1_, was performed with 994 non-Hispanic white asthmatic subjects from three drug clinical trials: CAMP, CARE, and ACRN. Genotyping was performed on Affymetrix 6.0 arrays, and imputed data based on HapMap Phase 2, was used to measure the association of SNPs with AHR using a linear regression model. Replication of primary findings was attempted in 650 white subjects from DAG, and 3,354 white subjects from LHS. Evidence that the top SNPs were eQTL of their respective genes was sought using expression data available for 419 white CAMP subjects.

**Results:**

The top primary GWAS associations were in rs848788 (P-value 7.2E-07) and rs6731443 (P-value 2.5E-06), located within the *ITGB5* and *AGFG1* genes, respectively. The *AGFG1* result replicated at a nominally significant level in one independent population (LHS P-value 0.012), and the SNP had a nominally significant unadjusted P-value (0.0067) for being an eQTL of *AGFG1*.

**Conclusions:**

Based on current knowledge of *ITGB5* and *AGFG1,* our results suggest that variants within these genes may be involved in modulating AHR. Future functional studies are required to confirm that our associations represent true biologically significant findings.

## Background

Asthma is a common chronic respiratory disease affecting over 25 million Americans and its prevalence has risen over the past decade [1]. Airway hyperresponsiveness (AHR), in which the muscles of the airway contract in response to certain exposures, is one of the primary characteristics of asthma and one that has been correlated with current asthma severity and future lung function [2-4]. Bronchoprovocation challenges with methacholine or histamine have been widely used to quantify AHR in clinical and research settings [5]. These tests consist in administration of increasing dosages of a bronchoconstrictor until a specific decrease in lung function is measured (e.g., 20% drop in FEV_1_). The mechanisms by which AHR occurs are not fully understood, but AHR has been related directly to changes in airway smooth muscle contractility as well as inflammation and airway remodeling [6,7]. Asthma is a disease with demonstrated heritability in humans, and several genes, including the *IKZF3-ZPBP2-GSDMB-ORMDL3* locus, *HLA-DQ, IL1RL1, IL33, TSLP, SLC22A5, SMAD3,* and *RORA* have been consistently associated with asthma in genome-wide association studies (GWAS) [8,9]. Although AHR is often used as a quantifiable and reproducible surrogate of asthma in animal model studies of asthma genetics [10,11], the genetics of AHR have not been widely studied in humans. Nonetheless, the heritability of AHR is supported by a previous twin study [12] and positional cloning and linkage analysis studies of AHR [13,14]. Our goal was to measure the association of genetic variants with AHR severity via a GWAS.

We utilized data from 994 non-Hispanic white asthma patients to measure the association of single nucleotide polymorphisms (SNPs) with severity of AHR and found that the strongest associations were at variants in two genes: ArfGAP with FG repeats 1 (*AGFG1*) and integrin, beta 5 (*ITGB5*). After attempting to replicate primary findings in two independent populations (one composed of subjects with asthma and one composed of patients with COPD) and searching for evidence that variants modified expression levels of their respective genes, we found additional evidence to support the involvement of *AGFG1* in AHR. Evidence that *ITGB5* was involved in AHR was found in previous functional studies of human airway smooth muscle cells.

## Methods

### Subjects

The primary group of subjects consisted of 994 non-Hispanic white asthmatics from the Childhood Asthma Management Program (CAMP) [15], and subsets of clinical trials within the Childhood Asthma Research and Education (CARE) network [16], and the Asthma Clinical Research Network (ACRN) [17] participating in the NHLBI SNP Health Association Resource Asthma Resource project (SHARP). Some demographic characteristics of these cohorts are in Table 1 and further details are provided in the Additional file 1. For each trial, methacholine challenge tests were performed according to American Thoracic Society criteria [18]. Baseline AHR measures were utilized, and AHR was quantified as the natural log of the provocative concentration of methacholine that caused a 20% decrease in FEV_1_ (LnPC20).

**Table 1:** Characteristics of GWAS subjects

	**CAMP**	**CARE**^**§**^	**ACRN**^**¶**^	**LHS**	**DAG**^**|**^
	**(n = 525)**	**(n = 195)**	**(n = 274)**	**(n = 3,354)**	**(n = 650)**
Age - Mean (SD)	8.8 (2.1)	10.6 (2.9)	31.5 (10.3)	48.8 (6.7)	36.0 (13.8)
Gender – N (%) Male	322 (61)	123 (63)	118 (43)	1949 (58.1)	291 (45)
Wash-out Prior to Methacholine Test* - Weeks	4	0-4	1-6	-	-
LnPC20	0.096 (1.21)	−0.25 (1.35)	0.80 (0.68)	1.58 (0.93)	0.71 (1.63)
Current smokers	0 (0)	0 (0)	0 (0)	3,354 (100)	106 (16)
Baseline FEV_1_	1.7 (0.5)	2.2 (0.8)	3.2 (0.8)	2.7 (0.6)	2.8 (0.9)
Baseline FEV_1_ % Predicted	94.4 (14.1)	98.8 (13.0)	86.8 (13.5)	78.2 (10.0)	82.3 (20.5)
Height [cm]	132.8 (13.6)	143.4 (16.8)	170.3 (10.0)	171.3 (8.9)	172.1 (11.5)
Weight [kg]	32.4 (10.9)	42.9 (18.1)	75.6 (18.2)	75.1 (14.8)	74.7 (17.6)
BMI [kg/cm^2]	17.9 (3.1)	20.1 (4.9)	25.9 (5.3)	25.5 (3.9)	25.0 (4.8)

*Subjects were allowed to use rescue medication during wash-out period.

^§^CARE trials included: CLIC, PACT, MARS.

^¶^ACRN trials included: BAGS, DICE, IMPACT, PRICE.

^|^The LnPC20 of the DAG cohort was calculated for 600 of the 650 asthmatics in the analysis.

Mean (Standard deviation) reported unless otherwise noted. CAMP, CARE, ACRN, and DAG are composed of subjects with asthma and not COPD, while LHS is composed of subjects with COPD who did not have asthma at the time of enrollment but may have had a past history of asthma.

### Genotyping and quality control

Genome-wide SNP genotyping for SHARP subjects was performed by Affymetrix, Inc. (Santa Clara, CA) using the Affymetrix Genome-Wide Human SNP Array 6.0. Dataset quality control (QC) included the removal of related subjects (i.e. CAMP and CARE siblings), subjects with missingness >5%, SNPs with minor allele frequency (MAF) <0.05, missingness >5%, or not passing Hardy-Weinberg equilibrium based on a threshold of 1E-03. The genomic inflation factor (λ_GC_) for the remaining 442,036 SNPs genotyped in SHARP subjects was 1.007, demonstrating minimal population stratification. Further genotyping and QC measures are provided in the Additional file 1.

### Statistical analysis

Imputation of all SNPs available in HapMap Phase 2 Release 22 CEU data using the Markov Chain Haplotyping software (MaCH) [19] was performed for SHARP genotype data. The primary GWAS was based on the set of 2,154,322 imputed SNPs with MAF >0.05 and a ratio of empirically observed dosage variance to the expected (binomial) dosage variance greater than 0.3, indicating good quality of imputation. The association of SNPs with LnPC20 was measured with a linear regression model using dosage data as implemented in PLINK [20]. To be sure that demographic characteristics did not have a strong influence on the association results, an additional association model was created using sex, age, height, and study as covariates. Plots of association results near specific genes were created using LocusZoom with the hg18/HapMap Phase II CEU GenomeBuild/LD Population [21]. Combined P-values with replication populations were computed using Fisher’s combined probability method [22] where hypothesis tests in replication populations had one-sided alternatives, based on the direction of the association in SHARP, so that SNPs with association tests in opposite directions would not produce inappropriately small P-values. Replication of SNPs with P-values <1E-05 in the primary GWAS was attempted in two independent cohorts.

### Genome-wide gene expression data

The Asthma BRIDGE project collected mRNA expression level data from 2,520 mRNA microarrays on 210 Illumina HumanHT-12 chips, among which 218 arrays were genetic control arrays and 2,302 arrays were sample arrays from 1,481 subjects. We focused on the 419 arrays (47,053 gene probes) from whole blood samples of 419 white CAMP subjects utilized in the current study. Expression data were log2 transformed and quantile normalized. The R Bioconductor GGtools package (version 3.11.27) was used to perform cis-eQTL analysis, in which chi squared tests were used to assess whether genotypes of a SNP within 50 KB from both ends of a gene were associated with the expression levels of the gene after adjusting for age and gender [23].

### Replication study

Dutch Asthma GWAS (DAG). This cohort was comprised of 650 DAG subjects with doctor-diagnosed asthma and documented AHR [14,24]. All subjects had smoking history and steroid use data available at the time of the AHR test. Participants with known AHR but in remission during the test were excluded. Remission was defined as not on steroids and without 20% or greater fall in FEV_1_ during the AHR test. The AHR test was conducted using histamine or methacholine as a stimulus. AHR was quantified as the difference between FEV_1_ at baseline and at the dose step at which a 20% or greater FEV_1_ drop was achieved, divided by the dose of stimulant used (slope). Because two protocols were used, one with a 30-second tidal breathing method and a second with a 2-minute tidal breathing phase, the AHR slopes measured with the 30-second tidal breathing method were divided by 4, in order to compensate for the 4 times greater duration of administration of stimulus. Slope values were log-transformed so that they would follow a normal distribution. To compare DAG subjects to those of the primary GWAS, PC20 was calculated in 600 of the 650 subjects that had a 20% or greater drop in FEV_1_ at the highest provocation dose. Genotyping was performed using the Hapmap 317 K platform or Illumina 370 Duo Chip. Tests of AHR association were performed via linear regression, with smoking and inhaled/oral steroid use as covariates using PLINK.

Lung Health Study (LHS). This cohort consisted in a subset of 3,354 individuals with COPD from the multicenter (10 centers) North American LHS [25]. The initial study population consisted of 5,887 men and women (63% male) who were smokers (aged 35–60) at enrollment with spirometric evidence of mild to moderate lung function impairment. While a history of asthma was not a criterion for study exclusion, subjects were excluded if they regularly used asthma medications, including bronchodilators and glucocorticoids. For the AHR GWAS, the subset of European American LHS participants with adequate DNA and valid methacholine measurements at baseline was utilized. Spirometry was performed following ATS criteria, and details of the methacholine challenge tests have been provided previously [26]. Samples were genotyped using the Illumina Human660W-Quad v.1_A BeadChip. Genotyped SNPs were imputed from CEU using 1000 Genomes Project data as a reference. Association of SNPs with log10 of PC20 was measured using linear regression under an additive model and while adjusting for gender, age at baseline, clinic site, log10(weight in kg), FEV1 (in L), and FEV1/FVC.

The current study was approved by the Partners Human Research Committee (Partners HealthCare, Inc., Boston, MA). Collection of data for the existing human cohort studies was approved by the Institutional Review Board of the corresponding institution(s), which ensured that all procedures followed were in accordance with the ethical standards of the responsible committee on human experimentation, as detailed in the cited works. Informed consent was obtained for all study participants.

## Results

**Figure 1:**
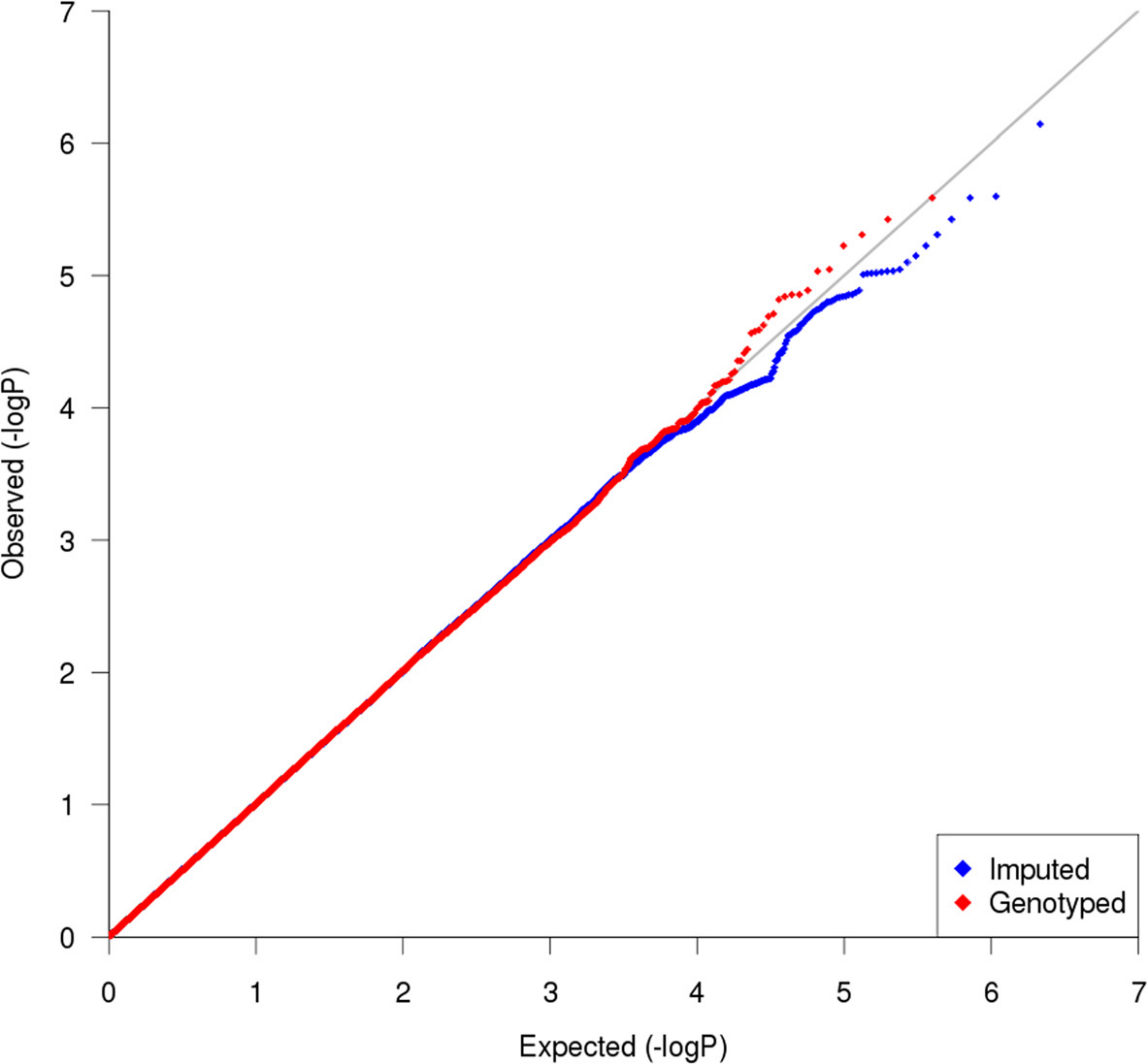
**Quantile-quantile (QQ) plot.** Comparison of primary GWAS P-values to those expected for a null distribution. Distribution of genotyped (red) and imputed (blue) GWAS results shows little evidence of deviation of measures at the tail, making the distinction among SNPs having low P-values representing true associations vs. those SNPs having low P-values by chance challenging.

**Figure 2:**
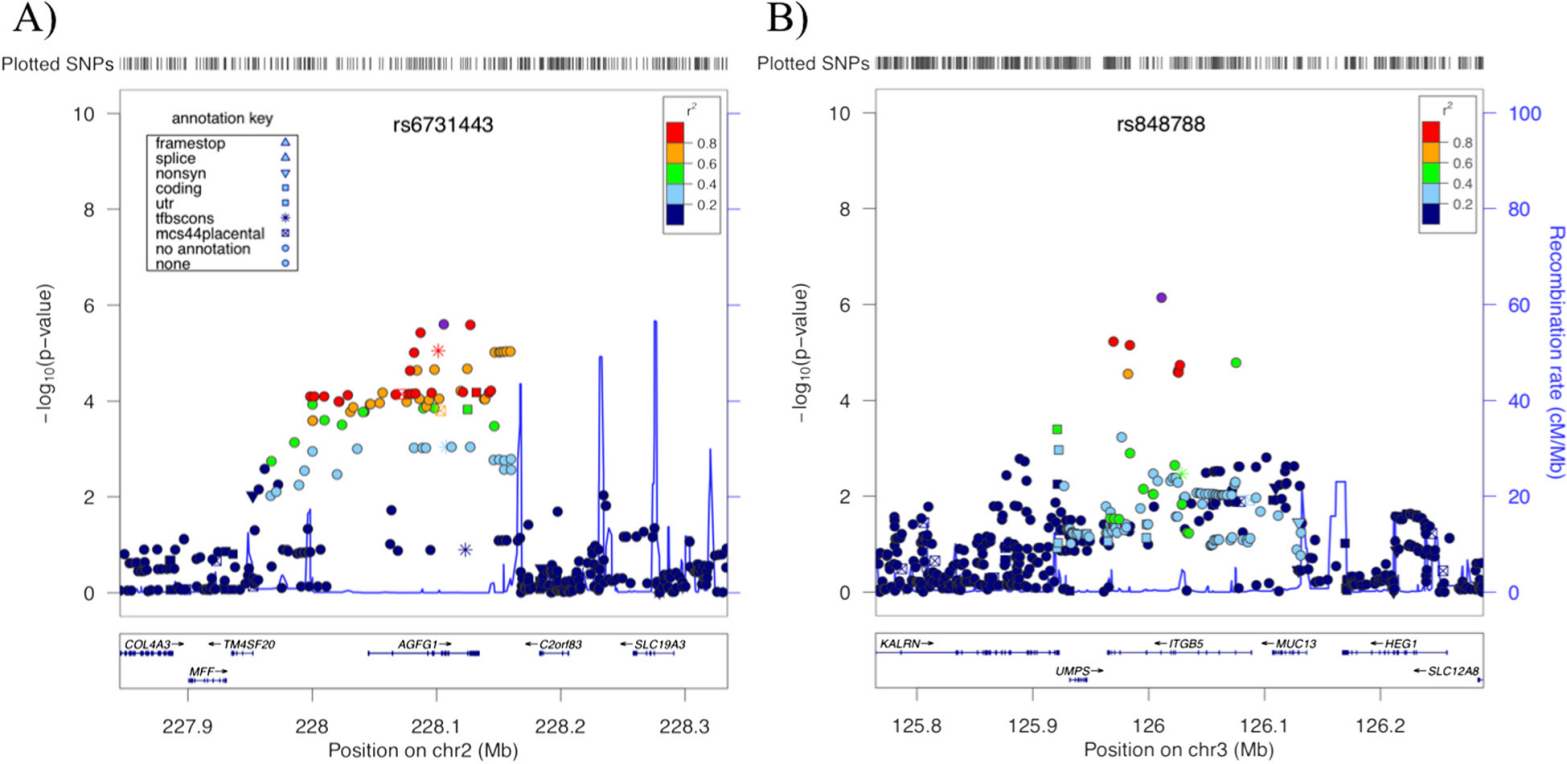
**Association regions of SNPs in/near (A) *AGFG1 *and (B) *ITGB5*.** The x-axes denote position along corresponding chromosomes. The y-axes denote –Log_10_(P) corresponding to primary GWAS P-values of association with AHR. LD between the SNPs with the lowest P-values (rs6731443 and rs848788, in panels **(A)** and **(B)**, respectively) to each SNP in the plots is denoted by colors and was computed according to hg18/HapMap Phase II CEU data. Plots were created using LocusZoom [21].

**Figure 3:**
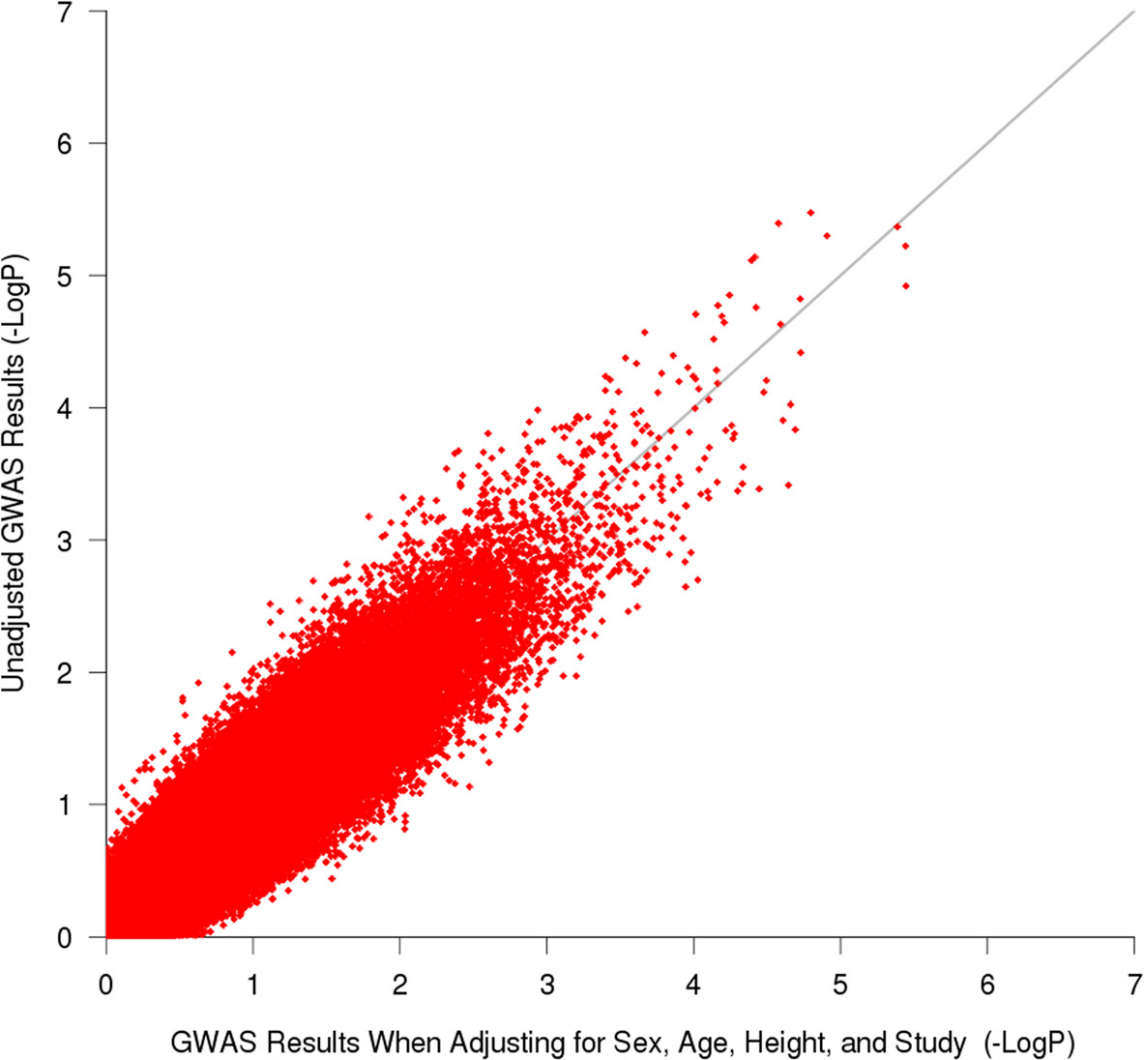
**Comparison of P-values generated for genotyped SNPs from the unadjusted primary GWAS vs. those from a model in which sex, age, height, and study were used as covariates.** The results reveal that adjustment for these covariates did not drastically alter the ranking or P-values of SNPs.

Characteristics of all subjects are provided in Table 1. The primary GWAS measured the association of SNPs with AHR in 994 non-Hispanic white subjects. The quantile-quantile (QQ) plots for both imputed and genotyped SNPs revealed that the distributions of association P-values were similar to those expected for a null distribution [Figure 1]. The lowest P-values (<1E-05; Table 2) among the primary GWAS are in four regions, two of which correspond to genes: ArfGAP with FG repeats 1 (*AGFG1*) and integrin, beta 5 (*ITGB5*) [Figure 2; Additional file 1: Table S1]. Comparison of primary association results to those generated with a model that adjusted for sex, age, height, and study revealed that there were no great changes in rankings or P-values, particularly for the top SNPs [Figure 3]. Because some of the top-ranked associations could represent biologically meaningful ones, we proceeded to attempt to replicate them in two independent populations. The SNPs in/near *AGFG1* replicated at a nominally significant level (i.e. P-value <0.05) in LHS, while no replication was obtained in DAG [Table 3].

**Table 2:** Primary GWAS top results (P-values <1E-05)

**SNP**	**CHR**	**BP**	**Reference allele**	**Frequency**	**Rsq**	**BETA**	**SE**	**P-value**	**Gene**
rs848788	3	126011114	C	0.36	0.97	−0.28	0.06	7.2E-07	*ITGB5*
rs6731443	2	228105810	G	0.55	1.00	−0.25	0.05	2.5E-06	*AGFG1*
rs13382948	2	228127201	A	0.45	1.00	0.25	0.05	2.6E-06	*AGFG1*
rs10189795	2	228087105	C	0.45	1.00	0.25	0.05	3.8E-06	*AGFG1*
rs4861175	4	41932655	A	0.18	1.00	−0.32	0.07	4.9E-06	*-*
rs614664	3	125969683	A	0.37	1.00	−0.25	0.06	6.0E-06	*ITGB5*
rs702036	3	125983762	A	0.63	0.98	0.25	0.06	7.1E-06	*ITGB5*
rs296282	5	106163683	C	0.22	0.56	0.38	0.09	8.0E-06	*-*
rs4972983	2	228101412	A	0.57	1.00	−0.24	0.05	9.0E-06	*AGFG1*
rs7592613	2	228159434	C	0.61	1.00	−0.24	0.05	9.3E-06	*AGFG1*
rs11683662	2	228156272	A	0.61	1.00	−0.24	0.05	9.3E-06	*AGFG1*
rs7579157	2	228153974	C	0.39	1.00	0.24	0.05	9.4E-06	*AGFG1*
rs7595130	2	228151796	A	0.39	1.00	0.24	0.05	9.5E-06	*AGFG1*
rs11674314	2	228150452	C	0.39	1.00	0.24	0.05	9.6E-06	*AGFG1*
rs4129709	2	228146793	A	0.61	1.00	−0.24	0.05	9.7E-06	*AGFG1*
rs4485561	2	228081927	G	0.43	1.00	0.24	0.05	9.8E-06	*AGFG1*

**Table 3:** Attempted replication of top results in independent populations

	**SHARP**	**LHS**		**DAG**			
**SNP**	**P-value**	**Beta**	**P-value**	**Beta**	**P-value**	**Fisher combined P-value**	***AGFG1 *eQTL P-value in CAMP**
rs6731443	2.5E-06	−0.022	0.012	−0.026	0.39	2.14E-06	0.0067
rs13382948	2.6E-06	0.021	0.015	0.026	0.39	2.75E-06	0.0067
rs10189795	3.8E-06	0.022	0.012	0.030	0.37	2.97E-06	0.0052
rs4972983	9.0E-06	−0.021	0.017	−0.034	0.35	8.62E-06	0.0052
rs4485561	9.8E-06	0.020	0.022	0.034	0.35	1.15E-05	0.0052
rs848788	7.2E-07	−0.009	0.20	0.027	0.61	1.30E-05	-
rs11683662	9.3E-06	−0.020	0.025	0.043	0.68	2.21E-05	-
rs7579157	9.4E-06	0.019	0.025	−0.041	0.67	2.23E-05	-
rs7595130	9.5E-06	0.019	0.025	−0.040	0.67	2.24E-05	-
rs7592613	9.3E-06	−0.020	0.026	0.042	0.68	2.25E-05	-
rs4129709	9.7E-06	−0.019	0.025	0.045	0.69	2.27E-05	0.034
rs11674314	9.6E-06	0.019	0.025	−0.041	0.68	2.27E-05	0.034
rs614664	6.0E-06	−0.011	0.14	0.041	0.67	6.73E-05	-
rs702036	7.1E-06	0.011	0.14	−0.037	0.65	7.58E-05	-
rs4861175	4.9E-06	0.003	0.60	0.077	0.92	2.60E-04	-
rs296282	8.0E-06	0.001	0.46	-	-	-	-

Reference alleles are those listed in Table 2. For eQTL, the gene for which transcripts are differentially expressed based on the corresponding SNP is *AGFG1*. SNPs with no listed eQTL P-values do not meet nominal significance (i.e. P-value >0.05) for any gene.

**Figure 4:**
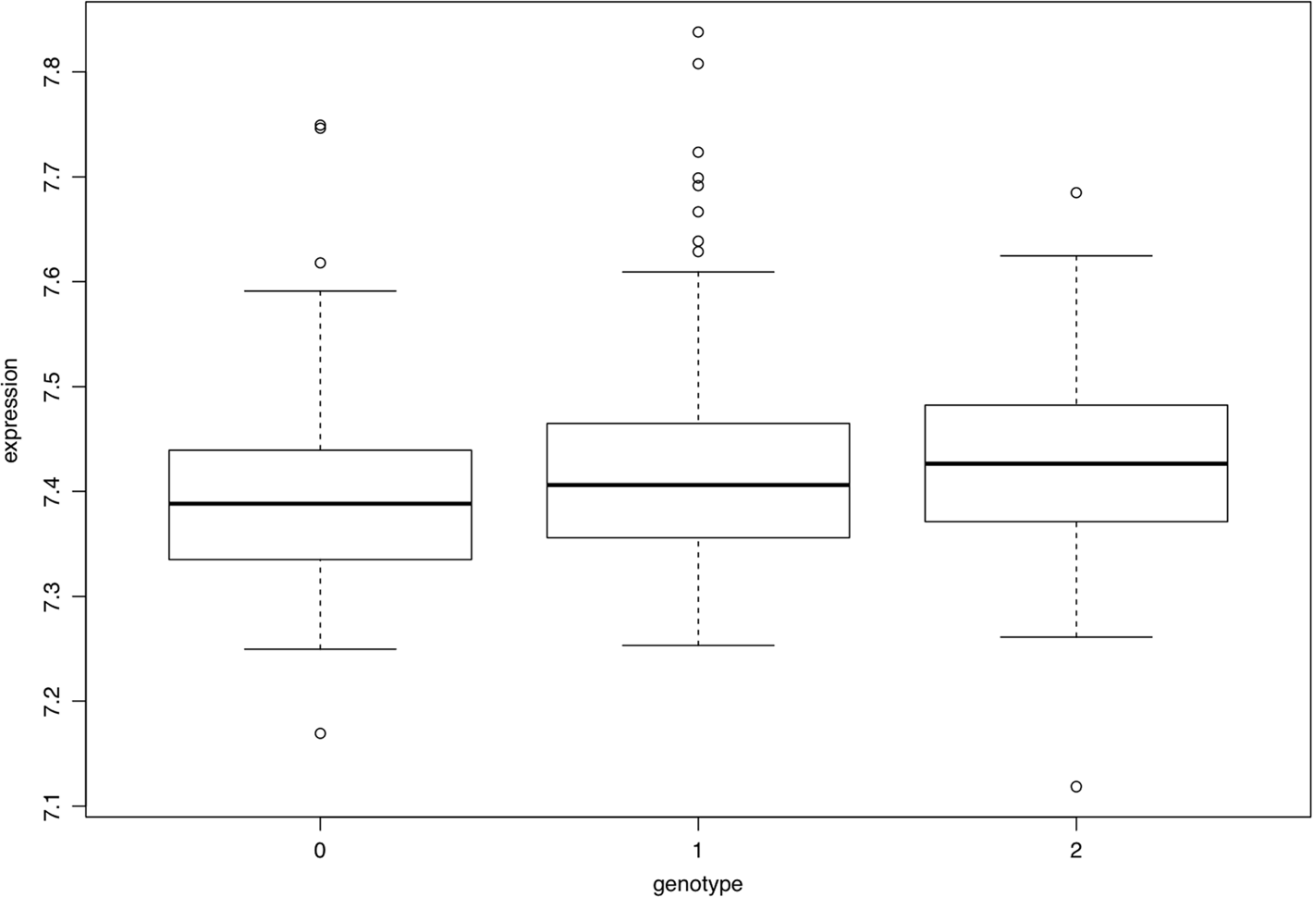
**Expression Quantitative Trait Loci (eQTL) results for *AGFG1 *expression levels by genotype of SNP rs6731443.** The genotypes represented along the x-axis are GG (0), GT (1) and TT (2). The AGFG1 probe used to plot expression levels has Illumina Probe ID Hoirhennr8UexQTd6I. Plots of *AGFG1* eQTLs for other top SNPs in Table 2 are similar to those in this Figure.

To find further support that the associations measured represent biologically relevant findings, we searched for evidence that any are eQTL for the genes in/near them among CAMP subjects who were part of the Asthma BRIDGE genomics study. The SNPs in/near *AGFG1* had nominally significant unadjusted P-values for being associated with expression levels of *AGFG1* in whole blood [Table 3, Figure 4]. None of these P-values passed Benjamini-Hochberg correction criteria as part of a genome-wide search for eQTL, nor were the expression levels of *AGFG1* significantly correlated with AHR. None of the other SNPs with P-value <1E-05 in the primary GWAS had nominally significant eQTL.

## Discussion

Our understanding of asthma genetics has increased in recent years, partly because of the success of large-scale multi-center GWAS [8,9]. Asthma GWAS have identified a robust set of genes that are consistently associated with the disease in diverse populations (e.g. the *IKZF3-ZPBP2-GSDMB-ORMDL3* locus, *TSLP,* and *RORA*), as well as genes that seem to be race/ethnicity specific (e.g. *PYHIN1*). In addition to studying asthma directly, GWAS have been carried out for related traits, including bronchodilator response [27], plasma IgE concentration [28], and response to inhaled corticosteroids [29]. Compared to asthma GWAS, the study of asthma-related traits has been challenged by the smaller sized cohorts available and the lack of uniform measures of interest across cohorts. While asthma-related trait GWAS often suffer from decreased statistical power due to their smaller sample sizes, they have some advantages over the study of asthma. Asthma-related traits can be expressed quantitatively, and this may result in increased statistical power to detect associations by capturing phenotypes that do not require arbitrary classification thresholds [30]. Moreover, the investigation of specific intermediate phenotypes of asthma may reduce phenotypic heterogeneity and facilitate the functional validation of association results as some of these traits are more easily simulated *in vitro* and in animal models [27].

Because AHR is a complex phenotype, our GWAS results are limited by our ability to properly define it in subjects. In a previous study of CAMP subjects, the repeatability of PC20 measures over the 4-year length of the clinical trial was high, as demonstrated by intraclass correlation coefficients, after adjusting for age, race/ethnicity, height, family income, parental education, and symptom score, of 0.67 for subjects taking budesonide or placebo and 0.73 for subjects taking nedocromil [31]. A twin study of methacholine responsiveness found that the intrapair correlation coefficient among monozygotic twins (0.67) was significantly greater than that for dizygotic twins (0.34) [12]. Positional cloning and linkage analysis studies of AHR have identified two well-validated asthma candidate genes: *ADAM33*[13] and *PCDH1*[14]. Although the high intraclass correlation of AHR measures and previous twin and association studies suggest that AHR is a heritable trait, AHR varies in time within individuals and is influenced by environmental factors and medication usage. Among the baseline AHR measures used in our GWAS, some were taken after placebo washout periods of varying length, while others were taken in subjects (e.g. some from CARE) who were not necessarily off of asthma medications [Table 1]. This difference would decrease our ability to detect significant relationships, such that less significant associations would be detected than if all subjects had had a placebo washout.

We performed our GWAS using subjects with AHR measures from as many asthma trials as possible in order to maximize our statistical power to detect associations. Nonetheless, partly because AHR is not always measured in asthma clinical trials, our sample size was limited to 994. One significant difference among the trials we utilized is that the CAMP inclusion criteria included having AHR, such that 12.5 mg/dl of methacholine or less resulted in a 20% drop in FEV_1_. Nonetheless, CAMP and CARE had similarly severe AHR (mean LnPC20 0.096 (SD 1.21) and −0.25 (SD 1.35), respectively). CAMP and CARE were also similar in terms of age, sex, and baseline FEV_1_ [Table 1]. These two pediatric populations contrast with ACRN, which was composed mostly of adults, had a greater proportion of female participants, less severe AHR (mean LnPC20 0.80 (SD 0.68)), and lower baseline FEV_1_. We investigated some of the effects of trial heterogeneity on our primary association results by performing the GWAS while adjusting for sex, age, height, and study. As Figure 3 shows, these covariates did not have a strong influence on the ranks or P-values obtained. Because performing the adjustment for covariates limited our sample size further to 989 due to missing demographic variable entries for some subjects, we opted to utilize the unadjusted results.

The replication populations were also distinct from our primary populations. DAG quantified AHR differently than all other cohorts, as the slope defined by the change in FEV_1_ between the stimulant dose step at which a 20% or greater FEV_1_ drop was achieved vs. baseline, divided by the dose of stimulant used. This definition allowed for the inclusion of subjects who did not achieve a PC20 at the doses of stimulants administered, and hence, allowed for the inclusion of subjects with less severe AHR. Fifty of 650 DAG subjects did not have PC20 measures, and hence, the estimate of mean LnPC20 provided in Table 1 is biased in that it does not include the measures of patients with less severe AHR. The AHR severity among LHS participants was also lower, on average, than that for primary cohort subjects (mean LnPC20 1.58 (SD 0.93)). While no participants of CAMP, CARE, or ACRN were smokers at the time of baseline AHR measures, LHS was a clinical trial that specifically enrolled adult smokers with COPD, while DAG was an observational study that included both smokers and non-smokers with asthma. Because smoking is known to affect lung function, AHR measures in both of these cohorts are likely affected by the smoking status of their participants. Further, LHS was not composed of asthma patients, and hence, it is likely that some of the biological mechanisms underlying its subjects AHR do not overlap with those of asthma patients. Despite the large differences among cohorts, we attempted to replicate our primary findings in LHS and DAG to assess their generalizability.

For the primary GWAS, four independent regions had P-values <1E-05 [Table 2]. While due to the small sample size of our primary GWAS, it is not surprising that none of these regions met traditional genome-wide significance levels, two of the top regions were composed of multiple SNPs within two genes (i.e. *AGFG1* and *ITGB5*), which increased the likelihood that the associations were biologically significant. The top *ITGB5* association was at SNP rs848788 (P-value 7.2E-07), while the top *AGFG1* association was at SNP rs6731443 (P-value 2.5E-06), both intronic SNPs of the corresponding genes. We utilized the AceView tool [32] to gather current information for these genes. The *ITGB5* gene maps to chromosome 3 at 3q21.2, and covers 139.46 kb, from 124620254 to 124480791 (NCBI 37, August 2010). *ITGB5* is a highly expressed gene in many tissues, including lung, with 15 different mRNAs and 10 probable alternative promoters. The gene has been studied widely and its product, integrin β5, is involved in cell adhesion and integrin-mediated signaling. Of most relevance to AHR, one study by Tatler, et al., investigated whether contraction agonists could promote TGF-β activation in human airway smooth muscle (HASM) cells and found that integrin ανβ5 was a mediator of this activation [33]. Specifically, lysophosphatidic acid (LPA) and methacholine activation of TGF-β, a cytokine that has been implicated in airway remodeling in asthma, was shown to be abrogated by an ανβ5 blocking antibody. Further, the β5 cytoplasmic domain of integrin ανβ5 was found to be involved in LPA activation because a polymorphism in this subunit reversed the integrin ανβ5 activation of TGF-β. Comparison of normal and asthmatic HASM cells found that asthmatic HASM had increased LPA-induced, integrin ανβ5-mediated TGF-β activity, and that this increase was not due to increased cell surface expression of integrin ανβ5. While we were unable to replicate the primary GWAS *ITGB5* associations in two independent populations, and there was no evidence that the corresponding SNPs were eQTL in whole blood of CAMP subjects, the study by Tatler, et al., suggests that polymorphisms in *ITGB5* could play a role in modulating HASM response to contraction agonists via mechanisms that do not involve changes to the expression levels of the genes whose products form integrin ανβ5. Further, it is possible that our top *ITGB5* SNPs are eQTLs of this gene HASM cells and not in whole blood, which is the tissue for which we currently have eQTL measures. Thus, it is possible that the *ITGB5* association we observed truly reflects a genetic change that modulates AHR.

Our association results for *AGFG1* variants were stronger than for *ITGB5* ones in that we were able to replicate them in one of two independent populations (lowest combined P-value across all cohorts was rs6731443 2.14E-06) and the corresponding SNPs were nominally significant eQTL of *AGFG1*. The *AGFG1* gene maps to chromosome 2, at 2q36.3, covering 89.07 kb, from 228336873 to 228425938 (NCBI 37, August 2010). *AGFG1* is also a highly expressed gene in many tissues, including lung, with 17 different mRNAs and 4 probable alternative promoters. The protein encoded by this gene (a.k.a. *RIP*, *HRB*) binds the activation domain of the human immunodeficiency virus (HIV) Rev protein when Rev is assembled onto its RNA target, and is required for the nuclear export of Rev-directed RNAs [34]. While many studies of *AGFG1* focus on its relationship to HIV, *AGFG1* has also been found to be involved in influenza A genome trafficking from the host nucleus to plasma membrane [35]. Because asthma development and severity are known to be influenced by respiratory pathogens, including influenza [36], current knowledge of *AGFG1* suggests that if our association and eQTL data for this gene represent true biologically significant findings, their relationship with AHR may involve changes in immune response or susceptibility to external factors (e.g., influenza). Further functional validation of the eQTL results, including via quantitative RT-PCR of *AGFG1* in subjects with various genotypes of the SNPs listed in Table 3 and for various tissues/cell types, would help clarify whether any relationship between the identified *AGFG1* variants and its expression levels truly exists. Because the nominal replication of *AGFG1* associations was observed in LHS, a clinical trial that measured baseline AHR in smokers who did not have current asthma, it is possible that the *AGFG1* association reflects biological processes modulating AHR that are not unique to asthma.

## Conclusions

An AHR GWAS among 994 asthma patients found that the most strongly associated SNPs, rs848788 (P-value 7.2E-07) and rs6731443 (P-value 2.5E-06), were in the *ITGB5* and *AGFG1* genes, respectively. The *ITGB5* association did not replicate in two independent populations, nor was there any evidence that the corresponding SNP was an eQTL of *ITGB5*. The *AGFG1* result replicated at a nominally significant level in one independent population of COPD subjects (LHS P-value 0.012), and the SNP had a nominally significant unadjusted P-value (0.0067) for being eQTL of *AGFG1*. While future functional studies are required to validate the potential involvement of these SNPs in modulating AHR, current knowledge of both genes suggests that our associations may represent biologically significant findings.

## Abbreviations

ACRN: Asthma Clinical Research Network; ADAM33: ADAM metallopeptidase domain 33; AGFG1: ArfGAP with FG repeats 1; AHR: Airway hyperresponsiveness; CAMP: Childhood Asthma Management Program; CARE: Childhood Asthma Research and Education; DAG: Dutch Asthma GWAS; eQTL: Expression quantitative trait loci; FEV1: Forced expiratory volume in one second; FVC: Forced vital capacity; GWAS: Genome-wide association study; GSDMB: Gasdermin B; HASM: Human airway smooth muscle; HLA-DQ: Major histocompatibility complex, class II, *DQ*; IKZF3: IKAROS family zinc finger 3 (Aiolos); IL1RL1: Interleukin 1 receptor-like 1; IL33: Interleukin 33; ITGB5: Integrin, beta 5; LHS: Lung Health Study; LnPC20: Natural log of methacholine concentration causing a 20% decrease in FEV_1_; LPA: Lysophosphatidic acid; MAF: Minor allele frequency; ORMDL3: ORM1-like 3 (S. cerevisiae); PCDH1: Protocadherin 1; PYHIN1: Pyrin and HIN domain family, member 1; QC: Quality control; QQ: Quantile-quantile; RORA: RAR-related orphan receptor A; SHARP: NHLBI SNP Health Association Resource Asthma Resource project; SLC22A5: Solute carrier family 22 (organic cation/carnitine transporter), member 5; SMAD3: SMAD family member 3; SNPs: Single nucleotide polymorphisms; TSLP: Thymic stromal lymphopoietin; ZPBP2: Zona pellucida binding protein 2.

## Competing interests

The authors declare that they have no competing interests.

## Authors’ contributions

BEH designed the study, analyzed primary data, and drafted the manuscript. BR, KGT, and STW contributed to the overall study concept and design. WQ provided statistical support. BK, JZ, and JS processed genetic data of primary populations. SJS, RFL, RSZ, RCS, FDM, HB, VMC, EI, and DM collected subjects for and designed primary cohort clinical trials. GHK, MAEN, DSP and JMV conducted the association study in DAG. NR, NNH, KB conducted the association study in LHS. All authors edited the manuscript and approved its final draft.

## Pre-publication history

The pre-publication history for this paper can be accessed here:

http://www.biomedcentral.com/1471-2350/14/86/prepub

## Supplementary Material

Additional file 1 Online supplement. Click here for file
