# Supplementary material for: ITGB5 and AGFG1 variants are associated with severity of airway responsiveness
Source: BMC Med Genet. 2013 Aug 28;14:86. doi: 10.1186/1471-2350-14-86 (PMC3765944; doi:10.1186/1471-2350-14-86)
Supplement: Additional file 1 — Online supplement. [file 1471-2350-14-86-S1.doc]

**Online Supplement**

**Subjects**

1. **Childhood Asthma Management Program (CAMP).** This population is composed of non-Hispanic white subjects from a clinical trial that followed 1,041 asthmatic children for four years and nearly 80% of the original participants for 12 years^1^. Stringent inclusion criteria ensured that participants had mild to moderate asthma, which was assessed as having asthma symptoms at least twice per week, using asthma medication daily, or using an inhaled bronchodilator twice per week for six or more months of the year prior to recruitment. CAMP subjects had increased airway responsiveness, as established by a 20% or greater decrease in FEV_1_ after administration of up to 12.5mg/dl of methacholine. Airway responsiveness was determined by the decrease in FEV_1_ after administration of increasing concentrations of methacholine using the Wright nebuizer–tidal breathing technique. CAMP-certified pulmonary function technicians performed the test in accordance with protocol^2^ at least 4 hours after the last use of a short-acting bronchodilator and at least 24 hours after the last use of a long-acting bronchodilator (if one is used under physician-discretion treatment). To minimize the effects of factors other than the study medication and the course of the asthma, staff did not determine airway responsiveness within 4 weeks of an upper respiratory tract infection or use of oral steroids, or if the FEV_1_ at baseline was <70% of predicted. Baseline airway responsiveness measures took place after a 4-week medication wash-out period. CAMP participants and their parents provided DNA for genetic studies. Informed consent was obtained from all CAMP participants and their parents. The studies were approved by the Institutional Review Board of Partners’ Healthcare and by all eight CAMP clinical centers and the CAMP Data Coordinating Center.
2. **Asthma Clinical Research Network (ACRN).** The ACRN was established in 1993 by the National Heart, Lung and Blood Institute (NHLBI) to conduct multiple well designed clinical trials for rapid evaluation of new and existing therapeutic approaches to asthma and to disseminate laboratory and clinical findings to the health care community (http://www.acrn.org)^3, 4^. Adults patients from the following ACRN trials were used for the current study: Beta Agonist in Mild Asthma Study (BAGS; n=101)^5^, Dose of Inhaled Corticosteroids with Equisystemic Effects (DICE; n=36)^6^, Improving Asthma Control Trial (IMPACT; n=115)^7^, and Predicting Responses for Inhaled Corticosteroid Efficacy (PRICE; n=22)^8^. BAGS subjects were selected to have mild asthma, and after a six-week run-in on placebo, baseline airway responsiveness was measured. DICE subjects were selected to have mild-to-moderate asthma, be corticosteroid-naïve, and baseline airway responsiveness was measured at the time of recruitment. IMPACT subjects were selected to have mild persistent asthma, and after a four-week run-in on placebo, baseline airway responsiveness was measured. PRICE subjects were selected to have mild persistent asthma, and after a two-week run-in on placebo, baseline airway responsiveness was measured.
3. **Childhood Asthma Research and Education (CARE).** The CARE Network was established in 1999 by the NHLBI to evaluate treatments for children with asthma (http://www.asthma-carenet.org)^9-11^. Subjects from the following CARE trials were used for the current study: Characterizing the Response to a Leukotriene Receptor Antagonist and an Inhaled Corticosteroid (CLIC; n=45)^11^, Pediatric Asthma Controller Trial (PACT; n=132)^12^, and Montelukast or Azithromycin for Reduction of Inhaled Corticosteroids in Childhood Asthma (MARS; n=18)^13^. CLIC subjects were children selected to have mild to moderate persistent asthma, and baseline airway responsiveness was measured at the time of recruitment **off controller medications for at least 2-weeks**. PACT subjects were children selected to have mild to moderate persistent asthma, and after a 2-to-4-week run-in on placebo, baseline airway responsiveness was measured. MARS subjects were children selected to have moderate to severe persistent asthma, and were **on controller inhaled corticosteroids** when baseline airway responsiveness was measured.

**Data Quality Control**

Affymetrix, Inc. (Santa Clara, CA) performed the genotyping according to manufacturer’s protocol using the Affymetrix Genome-Wide Human SNP Array 6.0. Marker quality control (QC) was preformed on all autosomal markers, which were extracted from dbGAP for each of the 3 cohorts.

**1. CAMP.** Of 2,241 unique CAMP subjects genotyped on the Affymetrix 6.0 genotyping chip, 1,874 passed QC filters. Passing subjects all had greater than 95.5% completion rate (average completion rate was 99.4%). Genotypic genders, as determined by the PLINK^14^ sexcheck algorithm, agreed with stated gender. rgGRR^15^ was used to evaluate allele sharing between each subject pair, and subjects had the expected degree of relatedness, although a few “unrelated” subjects had possible cousin or avuncular relationships. Identity of each sample was confirmed by comparing up to 855 markers that overlap between the 6.0 chip and previous genotyping performed at the Channing Laboratory. In addition to the 2,241 unique subjects, replicate samples were run for 5 QC subjects: 81 samples for 4 CAMP subjects and 24 samples for 1 CEPH/HapMap subject. Observed average concordance among CAMP replicates was 99.36%, and observed concordance among the CEPH/HapMap replicates was 99.65%. Mendelian error rate among nuclear families, as evaluated with PLINK, had an average error rate of 0.10% per subject.

Of 934,940 SNP markers present on the 6.0 chip, 190,480 were failed for one or more of the following reasons: 1) probe sequences did not map uniquely to hg18 genome build, 2) P-value for Hardy-Weinberg equilibrium <10^-6^ in white or black founders, 3) completion rate <95%, 4) Mendel error count >3, 5) >1 discordant samples observed among all possible CEPH replicate pairs, and/or 6) >2 discordant samples among CAMP replicate pairs. The final number of passing markers in the dataset was 744,460 (79.6%). For the AHR GWAS, data corresponding to 525 non-Hispanic white subjects was used as these subjects had appropriate phenotype data available.

2. **ACRN**. Of 1,059 ACRN subjects genotyped on the Affymetrix 6.0 genotyping chip, 721 passed quality control (QC) filters. Passing subjects all had greater than 95% completion rate (average completion rate was 99.1%). Genotypic genders, as determined by PLINK’s sexcheck algorithm, agreed with stated genders. rgGRR was used to evaluate allele sharing between subject pairs, and all subjects had the expected degree of relatedness. Identity of each sample was confirmed by comparing up to 165 markers that overlap between the 6.0 chip and previous genotyping performed at the Channing Laboratory. In addition to the 1,059 ACRN subjects, 4 QC subjects were run with replicate samples. Observed average concordance among 24 replicate samples within the 6.0 dataset was 99.36%.

Of 934,940 SNP markers present on the 6.0 chip, 152,960 were failed for one or more of the following reasons: 1) probe sequences did not map uniquely to hg18 genome build, 2) P=value for Hardy-Weinberg equilibrium <10^-6^ in white or black founders, 3) completion rate <95%, or 4) >12 discordances observed among sample replicate pairs. The final number of passing markers in the dataset was 781,980 (83.6%). For the AHR GWAS, data corresponding to 274 non-Hispanic white subjects was used as these subjects had appropriate phenotype data available.

3. **CARE**. Of 1,604 unique CARE subjects run on the Affymetrix 6.0 genotyping chip, 1,550 passed quality control (QC) filters. Passing subjects all had greater than 96.5% completion rate (average completion rate was 99.2%). Genotypic genders, as determined by PLINK’s sexcheck algorithm, agreed with stated genders. Mendelian error rate among nuclear families, as evaluated with PLINK, had an average error rate of 0.14% per subject. rgGRR was used to evaluate allele sharing between subject pairs, and subjects found to have half-sib or avuncular relationships were given a relatedness warning flag in the subject information file.

Of 934,940 SNP markers present on the 6.0 chip, 186,890 were failed for one or more of the following reasons: 1) probe sequences did not map uniquely to hg18 genome build, 2) P-value for Hardy-Weinberg equilibrium <10^-6^ in white or black founders, 3) completion rate <95%, 4) Mendelian error count >3 among 344 nuclear families. The final number of passing markers in the dataset was 748,050 (80.0%). For the AHR GWAS, data corresponding to 195 non-Hispanic white subjects was used as these subjects had appropriate phenotype data available.

**Supplementary Table 1.** Primary GWAS results for all SNPs in or within 50kb of *ITGB5* (Chromosome 3 SNPs below) or *AGFG1* (Chromosome 2 SNPs below) listed by position along corresponding chromosome.

| SNP | CHR | BP | Al1 | Al2 | Freq1 | Rsq | BETA | SE | P-value |
| --- | --- | --- | --- | --- | --- | --- | --- | --- | --- |
| rs4673194 | 2 | 227996429 | A | G | 0.49 | 0.70 | 0.13 | 0.06 | 0.047 |
| rs13019848 | 2 | 227996783 | A | G | 0.09 | 0.99 | 0.14 | 0.09 | 0.14 |
| rs13019352 | 2 | 227996807 | A | G | 0.91 | 0.99 | -0.14 | 0.09 | 0.14 |
| rs11693569 | 2 | 227996908 | G | T | 0.91 | 0.99 | -0.14 | 0.09 | 0.14 |
| rs7557934 | 2 | 227997406 | A | G | 0.07 | 0.81 | 0.04 | 0.12 | 0.74 |
| rs11678643 | 2 | 227997904 | A | T | 0.09 | 0.99 | 0.14 | 0.09 | 0.14 |
| rs11695160 | 2 | 227998027 | C | T | 0.91 | 0.99 | -0.14 | 0.09 | 0.14 |
| rs11695410 | 2 | 227998338 | A | G | 0.09 | 0.99 | 0.14 | 0.09 | 0.14 |
| rs13431496 | 2 | 227998403 | C | G | 0.58 | 0.92 | -0.22 | 0.06 | 8.01E-05 |
| rs6711609 | 2 | 228000155 | G | T | 0.79 | 0.85 | -0.23 | 0.07 | 1.12E-03 |
| rs6753666 | 2 | 228000184 | C | T | 0.39 | 0.81 | 0.23 | 0.06 | 1.17E-04 |
| rs4293562 | 2 | 228000269 | C | T | 0.37 | 0.92 | 0.21 | 0.06 | 2.56E-04 |
| rs4395244 | 2 | 228000434 | C | G | 0.63 | 0.92 | -0.21 | 0.06 | 2.55E-04 |
| rs4470337 | 2 | 228001625 | A | G | 0.42 | 0.92 | 0.22 | 0.06 | 8.00E-05 |
| rs11688520 | 2 | 228004388 | C | T | 0.93 | 0.82 | -0.04 | 0.12 | 0.74 |
| rs13018139 | 2 | 228006666 | A | G | 0.05 | 0.79 | 0.21 | 0.14 | 0.12 |
| rs4675186 | 2 | 228009585 | C | T | 0.42 | 0.93 | 0.22 | 0.06 | 7.97E-05 |
| rs7589853 | 2 | 228009801 | A | C | 0.37 | 0.93 | 0.21 | 0.06 | 2.51E-04 |
| rs11680042 | 2 | 228011249 | A | G | 0.93 | 0.83 | -0.04 | 0.11 | 0.74 |
| rs11680045 | 2 | 228011266 | A | C | 0.93 | 0.83 | -0.04 | 0.11 | 0.74 |
| rs6436696 | 2 | 228019905 | C | T | 0.83 | 0.95 | -0.21 | 0.07 | 3.41E-03 |
| rs12616324 | 2 | 228021236 | C | T | 0.65 | 0.94 | -0.22 | 0.06 | 1.02E-04 |
| rs4441459 | 2 | 228021701 | G | T | 0.65 | 0.95 | -0.22 | 0.06 | 1.02E-04 |
| rs6708202 | 2 | 228023916 | C | T | 0.70 | 0.99 | -0.21 | 0.06 | 3.12E-04 |
| rs4675191 | 2 | 228028564 | A | C | 0.58 | 0.95 | -0.22 | 0.06 | 7.49E-05 |
| rs7563404 | 2 | 228030668 | A | T | 0.62 | 0.97 | -0.21 | 0.06 | 1.67E-04 |
| rs4673197 | 2 | 228032919 | C | T | 0.38 | 0.98 | 0.21 | 0.05 | 1.35E-04 |
| rs4673199 | 2 | 228036162 | C | T | 0.18 | 1.00 | 0.23 | 0.07 | 9.93E-04 |
| rs12694734 | 2 | 228040803 | C | T | 0.72 | 1.00 | -0.22 | 0.06 | 1.69E-04 |
| rs6720355 | 2 | 228041902 | C | T | 0.28 | 1.00 | 0.22 | 0.06 | 1.66E-04 |
| rs4675198 | 2 | 228046451 | A | T | 0.38 | 0.99 | 0.21 | 0.05 | 1.16E-04 |
| rs4675199 | 2 | 228046897 | C | G | 0.62 | 0.99 | -0.21 | 0.05 | 1.15E-04 |
| rs7594606 | 2 | 228054219 | G | T | 0.62 | 1.00 | -0.21 | 0.05 | 1.09E-04 |
| rs4257394 | 2 | 228056553 | C | T | 0.38 | 0.97 | 0.22 | 0.06 | 6.67E-05 |
| rs6742120 | 2 | 228062686 | C | G | 0.92 | 0.93 | -0.17 | 0.10 | 0.096 |
| rs10933188 | 2 | 228063662 | C | T | 0.92 | 0.98 | 0.24 | 0.10 | 0.019 |
| rs4547524 | 2 | 228067144 | A | T | 0.38 | 1.00 | 0.22 | 0.05 | 7.31E-05 |
| rs4254497 | 2 | 228067154 | A | G | 0.62 | 1.00 | -0.22 | 0.05 | 7.28E-05 |
| rs13025709 | 2 | 228068841 | A | T | 0.09 | 0.99 | 0.14 | 0.09 | 0.13 |
| rs4496327 | 2 | 228071676 | C | T | 0.38 | 1.00 | 0.22 | 0.05 | 7.17E-05 |
| rs10182920 | 2 | 228075891 | G | T | 0.62 | 1.00 | -0.21 | 0.05 | 1.03E-04 |
| rs4246652 | 2 | 228077580 | C | T | 0.38 | 1.00 | 0.22 | 0.05 | 7.10E-05 |
| rs4281891 | 2 | 228078671 | A | G | 0.65 | 0.98 | -0.24 | 0.06 | 2.35E-05 |
| rs4075127 | 2 | 228080036 | A | G | 0.38 | 1.00 | 0.22 | 0.05 | 7.04E-05 |
| rs12466158 | 2 | 228081859 | C | T | 0.81 | 1.00 | -0.23 | 0.07 | 9.50E-04 |
| rs4485561 | 2 | 228081927 | G | T | 0.43 | 1.00 | 0.24 | 0.05 | 9.84E-06 |
| rs10933190 | 2 | 228083031 | A | G | 0.38 | 1.00 | 0.22 | 0.05 | 6.93E-05 |
| rs4508591 | 2 | 228084282 | C | T | 0.65 | 0.98 | -0.24 | 0.06 | 2.31E-05 |
| rs4246647 | 2 | 228086157 | G | T | 0.39 | 0.97 | 0.22 | 0.06 | 8.93E-05 |
| rs10189795 | 2 | 228087105 | C | T | 0.45 | 1.00 | 0.25 | 0.05 | 3.77E-06 |
| rs6705042 | 2 | 228088692 | C | T | 0.81 | 1.00 | -0.23 | 0.07 | 9.51E-04 |
| rs10191552 | 2 | 228089352 | C | T | 0.72 | 1.00 | -0.22 | 0.06 | 1.41E-04 |
| rs10185822 | 2 | 228091429 | A | G | 0.81 | 1.00 | -0.23 | 0.07 | 9.52E-04 |
| rs12987213 | 2 | 228091834 | C | T | 0.39 | 0.99 | 0.21 | 0.05 | 1.30E-04 |
| rs4973499 | 2 | 228093877 | G | T | 0.38 | 1.00 | 0.21 | 0.05 | 9.46E-05 |
| rs13035207 | 2 | 228095069 | C | T | 0.09 | 0.99 | 0.14 | 0.09 | 0.13 |
| rs7578871 | 2 | 228096035 | A | G | 0.38 | 1.00 | 0.22 | 0.05 | 6.71E-05 |
| rs4365454 | 2 | 228098223 | C | G | 0.72 | 1.00 | -0.22 | 0.06 | 1.40E-04 |
| rs4349340 | 2 | 228098330 | C | T | 0.65 | 0.98 | -0.24 | 0.06 | 2.22E-05 |
| rs4972983 | 2 | 228101412 | A | C | 0.57 | 1.00 | -0.24 | 0.05 | 9.01E-06 |
| rs7608104 | 2 | 228102067 | C | T | 0.38 | 1.00 | 0.21 | 0.05 | 8.90E-05 |
| rs6707385 | 2 | 228103250 | C | G | 0.60 | 0.96 | -0.21 | 0.05 | 1.58E-04 |
| rs6731443 | 2 | 228105810 | G | T | 0.55 | 1.00 | -0.25 | 0.05 | 2.52E-06 |
| rs7588620 | 2 | 228107724 | C | T | 0.82 | 1.00 | -0.23 | 0.07 | 9.08E-04 |
| rs10933191 | 2 | 228112047 | A | G | 0.82 | 1.00 | -0.23 | 0.07 | 9.08E-04 |
| rs10933192 | 2 | 228119431 | C | T | 0.62 | 0.98 | -0.22 | 0.05 | 6.11E-05 |
| rs4973181 | 2 | 228121240 | A | C | 0.62 | 1.00 | -0.22 | 0.05 | 6.46E-05 |
| rs4635528 | 2 | 228123218 | A | G | 0.91 | 0.99 | -0.14 | 0.09 | 0.13 |
| rs7598597 | 2 | 228124742 | C | T | 0.35 | 0.98 | 0.24 | 0.06 | 2.14E-05 |
| rs13426457 | 2 | 228124956 | A | G | 0.26 | 0.96 | 0.23 | 0.06 | 1.50E-04 |
| rs6761651 | 2 | 228127101 | C | G | 0.18 | 1.00 | 0.23 | 0.07 | 9.09E-04 |
| rs13382948 | 2 | 228127201 | A | G | 0.45 | 1.00 | 0.25 | 0.05 | 2.59E-06 |
| rs4312485 | 2 | 228132015 | C | G | 0.38 | 1.00 | 0.22 | 0.05 | 6.59E-05 |
| rs4246631 | 2 | 228138305 | G | T | 0.38 | 1.00 | 0.21 | 0.05 | 9.08E-05 |
| rs12619262 | 2 | 228139109 | C | T | 0.38 | 1.00 | 0.21 | 0.05 | 9.12E-05 |
| rs3924775 | 2 | 228142161 | G | T | 0.62 | 1.00 | -0.22 | 0.05 | 6.66E-05 |
| rs4420695 | 2 | 228143703 | C | T | 0.62 | 0.98 | -0.22 | 0.05 | 6.05E-05 |
| rs6738421 | 2 | 228146034 | C | G | 0.78 | 1.00 | -0.20 | 0.06 | 1.70E-03 |
| rs11676303 | 2 | 228146482 | C | T | 0.29 | 1.00 | 0.21 | 0.06 | 3.33E-04 |
| rs4129709 | 2 | 228146793 | A | G | 0.61 | 1.00 | -0.24 | 0.05 | 9.70E-06 |
| rs12987984 | 2 | 228149988 | C | T | 0.90 | 1.00 | -0.15 | 0.09 | 0.082 |
| rs4274592 | 2 | 228150405 | C | T | 0.78 | 1.00 | -0.20 | 0.06 | 1.68E-03 |
| rs11674314 | 2 | 228150452 | C | T | 0.39 | 1.00 | 0.24 | 0.05 | 9.63E-06 |
| rs7595130 | 2 | 228151796 | A | G | 0.39 | 1.00 | 0.24 | 0.05 | 9.55E-06 |
| rs12466978 | 2 | 228152917 | C | T | 0.90 | 1.00 | -0.15 | 0.09 | 0.081 |
| rs7579157 | 2 | 228153974 | C | T | 0.39 | 1.00 | 0.24 | 0.05 | 9.44E-06 |
| rs12328373 | 2 | 228154445 | A | G | 0.19 | 0.99 | 0.21 | 0.07 | 2.69E-03 |
| rs10933193 | 2 | 228154565 | G | T | 0.79 | 0.96 | -0.21 | 0.07 | 1.74E-03 |
| rs6734254 | 2 | 228155840 | C | T | 0.90 | 1.00 | -0.16 | 0.09 | 0.081 |
| rs11683662 | 2 | 228156272 | A | G | 0.61 | 1.00 | -0.24 | 0.05 | 9.30E-06 |
| rs12470596 | 2 | 228156351 | C | G | 0.90 | 1.00 | -0.16 | 0.09 | 0.081 |
| rs7592613 | 2 | 228159434 | C | T | 0.61 | 1.00 | -0.24 | 0.05 | 9.28E-06 |
| rs4973098 | 2 | 228159840 | A | G | 0.78 | 0.99 | -0.20 | 0.06 | 1.63E-03 |
| rs10204466 | 2 | 228160015 | A | G | 0.81 | 0.97 | -0.21 | 0.07 | 2.73E-03 |
| rs6752835 | 2 | 228165361 | G | T | 0.91 | 0.86 | -0.17 | 0.10 | 0.082 |
| rs7608226 | 2 | 228167161 | C | T | 0.87 | 0.83 | -0.15 | 0.09 | 0.072 |
| rs10933194 | 2 | 228167733 | C | T | 0.06 | 0.75 | 0.12 | 0.13 | 0.37 |
| rs6706866 | 2 | 228167964 | C | T | 0.85 | 0.73 | 0.00 | 0.09 | 0.99 |
| rs11694508 | 2 | 228168488 | G | T | 0.89 | 0.96 | -0.03 | 0.08 | 0.71 |
| rs11687220 | 2 | 228168901 | C | T | 0.89 | 0.99 | -0.03 | 0.08 | 0.74 |
| rs1109322 | 2 | 228169061 | A | G | 0.67 | 0.77 | -0.04 | 0.06 | 0.50 |
| rs17368509 | 2 | 228170705 | C | T | 0.89 | 0.99 | -0.02 | 0.08 | 0.77 |
| rs13012024 | 2 | 228171025 | C | G | 0.43 | 0.91 | -0.02 | 0.06 | 0.73 |
| rs13011882 | 2 | 228171152 | A | G | 0.89 | 0.99 | -0.02 | 0.08 | 0.79 |
| rs6726580 | 2 | 228171176 | A | C | 0.89 | 0.99 | -0.02 | 0.08 | 0.80 |
| rs6755748 | 2 | 228171404 | C | G | 0.11 | 0.99 | 0.02 | 0.08 | 0.80 |
| rs6755669 | 2 | 228171501 | C | T | 0.89 | 0.99 | -0.02 | 0.08 | 0.82 |
| rs6755969 | 2 | 228171554 | A | G | 0.31 | 0.80 | 0.01 | 0.06 | 0.87 |
| rs11692232 | 2 | 228172409 | A | C | 0.11 | 1.00 | 0.02 | 0.08 | 0.84 |
| rs11675880 | 2 | 228172495 | G | T | 0.11 | 1.00 | 0.02 | 0.08 | 0.84 |
| rs10207659 | 2 | 228173824 | A | G | 0.18 | 0.74 | 0.17 | 0.08 | 0.038 |
| rs10210075 | 2 | 228173933 | A | G | 0.71 | 0.89 | 0.04 | 0.06 | 0.47 |
| rs10175370 | 2 | 228173992 | C | T | 0.67 | 0.95 | 0.03 | 0.06 | 0.57 |
| rs6725452 | 2 | 228174231 | C | T | 0.30 | 0.83 | 0.01 | 0.06 | 0.85 |
| rs6714320 | 2 | 228175076 | A | G | 0.58 | 0.99 | 0.01 | 0.05 | 0.84 |
| rs4973122 | 2 | 228178688 | C | T | 0.31 | 0.99 | -0.02 | 0.06 | 0.76 |
| rs332508 | 3 | 125914950 | C | T | 0.42 | 0.88 | -0.05 | 0.06 | 0.36 |
| rs893830 | 3 | 125916240 | A | G | 0.15 | 0.92 | -0.17 | 0.08 | 0.029 |
| rs332506 | 3 | 125918578 | C | T | 0.62 | 0.83 | 0.03 | 0.06 | 0.61 |
| rs11711269 | 3 | 125919639 | A | G | 0.62 | 0.70 | 0.16 | 0.07 | 0.014 |
| rs151184 | 3 | 125919989 | C | T | 0.37 | 0.83 | 0.13 | 0.06 | 0.035 |
| rs4234221 | 3 | 125921019 | C | T | 0.87 | 0.82 | 0.14 | 0.09 | 0.12 |
| rs332512 | 3 | 125921129 | C | T | 0.33 | 0.81 | -0.23 | 0.06 | 4.02E-04 |
| rs3772800 | 3 | 125921276 | A | C | 0.81 | 0.74 | 0.22 | 0.08 | 5.65E-03 |
| rs332513 | 3 | 125921817 | A | T | 0.14 | 0.83 | -0.14 | 0.08 | 0.093 |
| rs1162 | 3 | 125922375 | A | G | 0.63 | 0.75 | 0.21 | 0.07 | 1.08E-03 |
| rs162740 | 3 | 125923927 | A | G | 0.74 | 0.91 | -0.12 | 0.06 | 0.051 |
| rs332515 | 3 | 125924147 | G | T | 0.60 | 0.98 | -0.08 | 0.05 | 0.14 |
| rs606552 | 3 | 125924191 | A | G | 0.74 | 0.92 | -0.12 | 0.06 | 0.051 |
| rs332516 | 3 | 125924385 | A | T | 0.27 | 0.81 | 0.15 | 0.07 | 0.022 |
| rs3736388 | 3 | 125926212 | C | T | 0.42 | 1.00 | 0.01 | 0.05 | 0.87 |
| rs333267 | 3 | 125926997 | C | T | 0.21 | 0.99 | -0.18 | 0.07 | 6.16E-03 |
| rs961077 | 3 | 125927289 | C | T | 0.40 | 1.00 | 0.08 | 0.05 | 0.14 |
| rs16835902 | 3 | 125928454 | C | G | 0.58 | 1.00 | -0.01 | 0.05 | 0.87 |
| rs16835912 | 3 | 125928594 | A | C | 0.60 | 1.00 | -0.08 | 0.05 | 0.14 |
| rs17282057 | 3 | 125928767 | C | T | 0.13 | 0.98 | -0.17 | 0.08 | 0.032 |
| rs9838750 | 3 | 125929210 | C | T | 0.17 | 1.00 | -0.14 | 0.07 | 0.049 |
| rs13075273 | 3 | 125929594 | A | G | 0.17 | 1.00 | -0.14 | 0.07 | 0.049 |
| rs12492095 | 3 | 125930646 | A | T | 0.17 | 1.00 | -0.14 | 0.07 | 0.048 |
| rs1440148 | 3 | 125931053 | A | G | 0.17 | 1.00 | -0.14 | 0.07 | 0.048 |
| rs2669919 | 3 | 125931620 | G | T | 0.93 | 0.57 | -0.25 | 0.14 | 0.067 |
| rs2279197 | 3 | 125931838 | A | G | 0.17 | 1.00 | -0.14 | 0.07 | 0.048 |
| rs2279198 | 3 | 125931899 | A | G | 0.40 | 1.00 | 0.08 | 0.05 | 0.13 |
| rs2279199 | 3 | 125931942 | C | T | 0.47 | 0.99 | 0.00 | 0.05 | 0.94 |
| rs1139538 | 3 | 125931981 | A | G | 0.40 | 1.00 | 0.08 | 0.05 | 0.13 |
| rs669736 | 3 | 125932507 | A | T | 0.38 | 0.99 | 0.12 | 0.06 | 0.032 |
| rs4678145 | 3 | 125932771 | C | G | 0.13 | 0.98 | -0.18 | 0.08 | 0.032 |
| rs9840078 | 3 | 125932974 | G | T | 0.80 | 0.95 | 0.11 | 0.07 | 0.096 |
| rs10934681 | 3 | 125933011 | C | T | 0.28 | 0.99 | 0.10 | 0.06 | 0.085 |
| rs9844948 | 3 | 125933754 | A | C | 0.17 | 1.00 | -0.14 | 0.07 | 0.057 |
| rs10934682 | 3 | 125934002 | G | T | 0.17 | 1.00 | -0.13 | 0.07 | 0.059 |
| rs9870260 | 3 | 125934849 | A | T | 0.17 | 1.00 | -0.13 | 0.07 | 0.060 |
| rs11916134 | 3 | 125935804 | A | G | 0.72 | 1.00 | -0.10 | 0.06 | 0.086 |
| rs10934683 | 3 | 125936073 | C | T | 0.70 | 1.00 | -0.09 | 0.06 | 0.14 |
| rs3772804 | 3 | 125936864 | A | G | 0.72 | 1.00 | -0.10 | 0.06 | 0.087 |
| rs16835929 | 3 | 125937904 | A | G | 0.83 | 1.00 | 0.13 | 0.07 | 0.061 |
| rs1801019 | 3 | 125939432 | C | G | 0.17 | 1.00 | -0.13 | 0.07 | 0.061 |
| rs694897 | 3 | 125941506 | C | G | 0.36 | 0.96 | 0.12 | 0.06 | 0.032 |
| rs3821535 | 3 | 125943121 | A | G | 0.28 | 1.00 | 0.10 | 0.06 | 0.086 |
| rs13146 | 3 | 125945498 | C | T | 0.83 | 1.00 | 0.13 | 0.07 | 0.062 |
| rs2242248 | 3 | 125946672 | G | T | 0.83 | 1.00 | 0.13 | 0.07 | 0.062 |
| rs11706118 | 3 | 125946832 | A | C | 0.30 | 1.00 | 0.09 | 0.06 | 0.14 |
| rs1979412 | 3 | 125948647 | A | G | 0.17 | 0.98 | -0.13 | 0.07 | 0.071 |
| rs10934685 | 3 | 125949210 | C | T | 0.72 | 1.00 | -0.10 | 0.06 | 0.086 |
| rs659441 | 3 | 125961384 | C | G | 0.37 | 0.99 | 0.11 | 0.06 | 0.039 |
| rs13082230 | 3 | 125961424 | A | G | 0.53 | 1.00 | 0.00 | 0.05 | 0.98 |
| rs13087417 | 3 | 125961902 | C | T | 0.72 | 1.00 | -0.10 | 0.06 | 0.085 |
| rs2055983 | 3 | 125963120 | A | G | 0.80 | 0.93 | 0.17 | 0.07 | 0.016 |
| rs9840461 | 3 | 125963456 | C | G | 0.83 | 1.00 | 0.13 | 0.07 | 0.060 |
| rs9878092 | 3 | 125963533 | A | G | 0.83 | 1.00 | 0.13 | 0.07 | 0.059 |
| rs14386 | 3 | 125963742 | A | G | 0.17 | 1.00 | -0.13 | 0.07 | 0.059 |
| rs849019 | 3 | 125964151 | A | T | 0.37 | 0.98 | 0.12 | 0.06 | 0.038 |
| rs2676 | 3 | 125964677 | A | G | 0.83 | 1.00 | 0.13 | 0.07 | 0.059 |
| rs2674 | 3 | 125964912 | C | T | 0.88 | 0.93 | 0.14 | 0.08 | 0.087 |
| rs585021 | 3 | 125965559 | C | T | 0.90 | 0.46 | 0.04 | 0.13 | 0.79 |
| rs10934691 | 3 | 125965746 | C | G | 0.77 | 0.78 | -0.12 | 0.07 | 0.11 |
| rs2291079 | 3 | 125965779 | C | G | 0.77 | 0.78 | -0.14 | 0.07 | 0.054 |
| rs6795142 | 3 | 125966387 | C | T | 0.16 | 1.00 | -0.15 | 0.07 | 0.039 |
| rs3772812 | 3 | 125966523 | C | T | 0.81 | 1.00 | 0.14 | 0.07 | 0.042 |
| rs3821536 | 3 | 125966642 | C | T | 0.17 | 0.91 | -0.15 | 0.07 | 0.045 |
| rs3772813 | 3 | 125966763 | C | T | 0.16 | 1.00 | -0.15 | 0.07 | 0.039 |
| rs1877568 | 3 | 125966877 | G | T | 0.80 | 1.00 | 0.15 | 0.07 | 0.029 |
| rs3772815 | 3 | 125967009 | A | G | 0.87 | 0.96 | 0.19 | 0.08 | 0.022 |
| rs2291081 | 3 | 125967925 | A | G | 0.84 | 1.00 | 0.15 | 0.07 | 0.038 |
| rs3772817 | 3 | 125968560 | A | G | 0.85 | 0.98 | 0.15 | 0.08 | 0.054 |
| rs3772818 | 3 | 125968716 | A | G | 0.84 | 1.00 | 0.15 | 0.07 | 0.038 |
| rs614664 | 3 | 125969683 | A | C | 0.37 | 1.00 | -0.25 | 0.06 | 5.96E-06 |
| rs6792936 | 3 | 125969790 | C | T | 0.84 | 1.00 | 0.15 | 0.07 | 0.038 |
| rs6768588 | 3 | 125970025 | A | G | 0.80 | 1.00 | 0.15 | 0.07 | 0.030 |
| rs10049380 | 3 | 125970133 | C | T | 0.20 | 1.00 | -0.15 | 0.07 | 0.030 |
| rs2291082 | 3 | 125970539 | A | G | 0.80 | 0.98 | 0.14 | 0.07 | 0.040 |
| rs2840061 | 3 | 125970957 | A | G | 0.16 | 1.00 | -0.15 | 0.07 | 0.038 |
| rs2035265 | 3 | 125971300 | G | T | 0.20 | 0.99 | -0.14 | 0.07 | 0.046 |
| rs12488326 | 3 | 125972778 | A | G | 0.80 | 1.00 | 0.13 | 0.07 | 0.058 |
| rs1980078 | 3 | 125974349 | C | T | 0.20 | 1.00 | -0.15 | 0.07 | 0.030 |
| rs4558801 | 3 | 125976118 | A | G | 0.84 | 1.00 | 0.15 | 0.07 | 0.038 |
| rs621209 | 3 | 125976720 | A | G | 0.21 | 0.99 | -0.22 | 0.06 | 5.86E-04 |
| rs13064025 | 3 | 125979167 | C | T | 0.15 | 0.96 | -0.15 | 0.08 | 0.051 |
| rs2083425 | 3 | 125979578 | G | T | 0.84 | 0.99 | 0.15 | 0.07 | 0.039 |
| rs3772825 | 3 | 125980296 | A | G | 0.16 | 1.00 | -0.15 | 0.07 | 0.039 |
| rs9879289 | 3 | 125980863 | A | G | 0.14 | 0.98 | -0.16 | 0.08 | 0.045 |
| rs2035264 | 3 | 125982188 | C | T | 0.32 | 0.99 | -0.24 | 0.06 | 2.84E-05 |
| rs702037 | 3 | 125983231 | C | G | 0.28 | 0.94 | 0.12 | 0.06 | 0.055 |
| rs702036 | 3 | 125983762 | A | G | 0.63 | 0.98 | 0.25 | 0.06 | 7.10E-06 |
| rs702035 | 3 | 125983906 | A | G | 0.10 | 0.69 | -0.22 | 0.11 | 0.043 |
| rs2619328 | 3 | 125983912 | A | G | 0.29 | 0.89 | -0.20 | 0.06 | 1.27E-03 |
| rs3821538 | 3 | 125992901 | A | G | 0.84 | 1.00 | 0.17 | 0.07 | 0.018 |
| rs4678160 | 3 | 125995464 | C | G | 0.23 | 1.00 | -0.17 | 0.06 | 7.12E-03 |
| rs1803825 | 3 | 125997998 | A | G | 0.80 | 0.99 | 0.12 | 0.07 | 0.074 |
| rs2291088 | 3 | 125998199 | A | G | 0.16 | 1.00 | -0.15 | 0.07 | 0.039 |
| rs3772832 | 3 | 125998566 | A | G | 0.80 | 0.85 | 0.15 | 0.07 | 0.039 |
| rs4678162 | 3 | 125999385 | A | G | 0.16 | 0.99 | -0.15 | 0.07 | 0.039 |
| rs9874118 | 3 | 125999483 | C | T | 0.12 | 0.94 | -0.14 | 0.08 | 0.10 |
| rs12487905 | 3 | 126003725 | C | T | 0.20 | 0.97 | -0.18 | 0.07 | 9.11E-03 |
| rs17282085 | 3 | 126003953 | A | G | 0.12 | 0.95 | -0.25 | 0.08 | 3.41E-03 |
| rs12695446 | 3 | 126004047 | A | G | 0.20 | 0.97 | -0.18 | 0.07 | 9.17E-03 |
| rs13314004 | 3 | 126004572 | A | G | 0.06 | 0.74 | -0.30 | 0.12 | 0.017 |
| rs4678164 | 3 | 126007761 | A | G | 0.17 | 0.97 | -0.20 | 0.07 | 4.82E-03 |
| rs848788 | 3 | 126011114 | C | T | 0.36 | 0.97 | -0.28 | 0.06 | 7.21E-07 |
| rs2086272 | 3 | 126018839 | C | T | 0.83 | 0.97 | 0.20 | 0.07 | 4.83E-03 |
| rs4678165 | 3 | 126020879 | A | G | 0.12 | 0.98 | -0.24 | 0.08 | 4.17E-03 |
| rs6769379 | 3 | 126022524 | C | G | 0.81 | 0.99 | 0.21 | 0.07 | 2.26E-03 |
| rs4678166 | 3 | 126023246 | C | T | 0.88 | 0.98 | 0.23 | 0.08 | 4.16E-03 |
| rs3755710 | 3 | 126023392 | A | T | 0.12 | 0.99 | -0.23 | 0.08 | 4.16E-03 |
| rs4678167 | 3 | 126023522 | C | T | 0.85 | 0.99 | 0.17 | 0.08 | 0.027 |
| rs6438855 | 3 | 126024946 | A | G | 0.85 | 0.99 | 0.17 | 0.08 | 0.027 |
| rs6438856 | 3 | 126025221 | C | T | 0.66 | 1.00 | 0.24 | 0.06 | 2.59E-05 |
| rs9860625 | 3 | 126025265 | C | T | 0.83 | 1.00 | 0.20 | 0.07 | 5.20E-03 |
| rs4678168 | 3 | 126025716 | C | G | 0.66 | 1.00 | 0.24 | 0.06 | 2.38E-05 |
| rs4678169 | 3 | 126025793 | A | C | 0.34 | 1.00 | -0.24 | 0.06 | 2.66E-05 |
| rs7624392 | 3 | 126026956 | C | T | 0.34 | 0.98 | -0.24 | 0.06 | 1.85E-05 |
| rs6771896 | 3 | 126028440 | A | G | 0.18 | 0.99 | -0.20 | 0.07 | 3.44E-03 |
| rs4677946 | 3 | 126028737 | A | G | 0.79 | 1.00 | 0.16 | 0.07 | 0.015 |
| rs4677947 | 3 | 126028748 | A | G | 0.21 | 0.99 | -0.16 | 0.07 | 0.015 |
| rs2076722 | 3 | 126030561 | C | T | 0.23 | 0.95 | 0.17 | 0.07 | 0.011 |
| rs16836078 | 3 | 126031289 | C | T | 0.13 | 1.00 | -0.20 | 0.08 | 0.010 |
| rs16836080 | 3 | 126031374 | A | G | 0.28 | 1.00 | 0.12 | 0.06 | 0.049 |
| rs3851349 | 3 | 126031826 | G | T | 0.17 | 0.94 | -0.19 | 0.07 | 0.011 |
| rs3863065 | 3 | 126032881 | G | T | 0.11 | 0.69 | 0.01 | 0.10 | 0.93 |
| rs3772840 | 3 | 126033054 | C | T | 0.56 | 0.97 | -0.10 | 0.05 | 0.058 |
| rs17236654 | 3 | 126033236 | A | G | 0.05 | 0.98 | -0.08 | 0.12 | 0.51 |
| rs4141663 | 3 | 126034657 | C | T | 0.56 | 0.97 | -0.10 | 0.05 | 0.059 |
| rs3772841 | 3 | 126036820 | A | C | 0.82 | 0.95 | 0.18 | 0.07 | 0.014 |
| rs3772843 | 3 | 126041431 | A | G | 0.18 | 1.00 | -0.19 | 0.07 | 8.58E-03 |
| rs3772845 | 3 | 126043580 | C | T | 0.80 | 1.00 | 0.14 | 0.07 | 0.033 |
| rs6438858 | 3 | 126044170 | C | G | 0.82 | 1.00 | 0.18 | 0.07 | 8.74E-03 |
| rs3772847 | 3 | 126045205 | C | T | 0.18 | 1.00 | -0.18 | 0.07 | 8.80E-03 |
| rs12490725 | 3 | 126046263 | C | T | 0.87 | 0.99 | 0.20 | 0.08 | 0.015 |
| rs9865359 | 3 | 126048917 | A | G | 0.82 | 1.00 | 0.18 | 0.07 | 8.95E-03 |
| rs17309224 | 3 | 126049494 | G | T | 0.81 | 0.99 | -0.20 | 0.07 | 3.26E-03 |
| rs848807 | 3 | 126051501 | C | T | 0.20 | 0.99 | -0.15 | 0.07 | 0.026 |
| rs3772850 | 3 | 126051657 | C | G | 0.82 | 1.00 | 0.18 | 0.07 | 8.96E-03 |
| rs3772851 | 3 | 126053508 | G | T | 0.43 | 0.99 | 0.09 | 0.05 | 0.11 |
| rs3772852 | 3 | 126053649 | C | G | 0.57 | 0.99 | -0.09 | 0.05 | 0.10 |
| rs3772853 | 3 | 126054909 | A | G | 0.43 | 0.99 | 0.09 | 0.05 | 0.10 |
| rs13074444 | 3 | 126055252 | A | C | 0.43 | 0.99 | 0.09 | 0.05 | 0.10 |
| rs10212596 | 3 | 126055305 | C | T | 0.18 | 1.00 | -0.18 | 0.07 | 9.00E-03 |
| rs1265649 | 3 | 126055340 | A | G | 0.20 | 1.00 | -0.16 | 0.07 | 0.015 |
| rs1265650 | 3 | 126055689 | A | G | 0.80 | 1.00 | 0.16 | 0.07 | 0.015 |
| rs1265651 | 3 | 126056095 | C | T | 0.19 | 1.00 | 0.21 | 0.07 | 3.07E-03 |
| rs10934693 | 3 | 126056516 | C | G | 0.43 | 1.00 | 0.09 | 0.05 | 0.083 |
| rs848792 | 3 | 126057032 | C | T | 0.19 | 1.00 | 0.21 | 0.07 | 3.05E-03 |
| rs848793 | 3 | 126057409 | C | T | 0.20 | 1.00 | -0.16 | 0.07 | 0.014 |
| rs9809420 | 3 | 126057551 | A | G | 0.82 | 1.00 | 0.18 | 0.07 | 9.23E-03 |
| rs848794 | 3 | 126057751 | A | G | 0.20 | 1.00 | -0.16 | 0.07 | 0.014 |
| rs3772856 | 3 | 126058100 | C | T | 0.82 | 1.00 | 0.18 | 0.07 | 9.29E-03 |
| rs1499961 | 3 | 126058333 | C | G | 0.57 | 1.00 | -0.09 | 0.05 | 0.082 |
| rs848795 | 3 | 126058972 | A | G | 0.19 | 1.00 | 0.21 | 0.07 | 2.99E-03 |
| rs848796 | 3 | 126059045 | C | T | 0.19 | 1.00 | 0.21 | 0.07 | 3.00E-03 |
| rs9858709 | 3 | 126060154 | C | T | 0.82 | 1.00 | 0.18 | 0.07 | 9.48E-03 |
| rs9878701 | 3 | 126060215 | C | T | 0.18 | 1.00 | -0.18 | 0.07 | 9.52E-03 |
| rs11928651 | 3 | 126060245 | C | T | 0.43 | 1.00 | 0.10 | 0.05 | 0.081 |
| rs9859090 | 3 | 126060436 | C | T | 0.82 | 1.00 | 0.18 | 0.07 | 9.52E-03 |
| rs6778643 | 3 | 126060516 | C | T | 0.57 | 1.00 | -0.10 | 0.05 | 0.081 |
| rs848798 | 3 | 126061585 | C | G | 0.19 | 1.00 | 0.21 | 0.07 | 3.03E-03 |
| rs13089681 | 3 | 126062699 | C | T | 0.82 | 1.00 | 0.18 | 0.07 | 9.52E-03 |
| rs3772860 | 3 | 126063667 | A | G | 0.18 | 1.00 | -0.18 | 0.07 | 9.52E-03 |
| rs6787638 | 3 | 126065009 | C | T | 0.18 | 1.00 | -0.18 | 0.07 | 9.52E-03 |
| rs10934695 | 3 | 126067409 | C | T | 0.82 | 1.00 | 0.18 | 0.07 | 9.52E-03 |
| rs4234222 | 3 | 126067477 | A | G | 0.18 | 1.00 | -0.18 | 0.07 | 9.52E-03 |
| rs848803 | 3 | 126068998 | C | T | 0.80 | 1.00 | 0.16 | 0.07 | 0.013 |
| rs9813129 | 3 | 126070607 | G | T | 0.57 | 1.00 | -0.10 | 0.05 | 0.081 |
| rs13091878 | 3 | 126071619 | G | T | 0.18 | 1.00 | -0.18 | 0.07 | 9.51E-03 |
| rs2127304 | 3 | 126071919 | A | G | 0.83 | 0.92 | 0.20 | 0.08 | 7.58E-03 |
| rs3772865 | 3 | 126071974 | A | C | 0.43 | 1.00 | 0.10 | 0.05 | 0.073 |
| rs1270666 | 3 | 126073205 | A | T | 0.20 | 1.00 | -0.16 | 0.07 | 0.013 |
| rs7632893 | 3 | 126074109 | A | G | 0.18 | 1.00 | -0.19 | 0.07 | 5.76E-03 |
| rs1017813 | 3 | 126074675 | C | T | 0.18 | 1.00 | -0.20 | 0.07 | 5.09E-03 |
| rs1017814 | 3 | 126074829 | C | G | 0.18 | 1.00 | -0.20 | 0.07 | 5.09E-03 |
| rs1265646 | 3 | 126075390 | A | G | 0.38 | 1.00 | -0.24 | 0.05 | 1.65E-05 |
| rs1265647 | 3 | 126075528 | C | T | 0.20 | 1.00 | -0.16 | 0.07 | 0.013 |
| rs2047574 | 3 | 126077493 | A | G | 0.81 | 1.00 | -0.21 | 0.07 | 2.41E-03 |
| rs1948696 | 3 | 126077645 | C | T | 0.64 | 1.00 | -0.01 | 0.06 | 0.83 |
| rs12631064 | 3 | 126078363 | C | G | 0.81 | 1.00 | -0.21 | 0.07 | 2.42E-03 |
| rs4502626 | 3 | 126079342 | A | G | 0.80 | 1.00 | 0.16 | 0.07 | 0.013 |
| rs10804563 | 3 | 126081189 | C | T | 0.32 | 0.85 | 0.12 | 0.06 | 0.058 |
| rs10804564 | 3 | 126081216 | A | G | 0.57 | 1.00 | -0.10 | 0.05 | 0.081 |
| rs9968182 | 3 | 126084662 | C | T | 0.57 | 1.00 | -0.10 | 0.05 | 0.081 |
| rs4422355 | 3 | 126085572 | C | G | 0.43 | 1.00 | 0.09 | 0.05 | 0.090 |
| rs1007857 | 3 | 126087379 | A | C | 0.13 | 0.99 | -0.20 | 0.08 | 0.012 |
| rs1007856 | 3 | 126087548 | A | G | 0.57 | 1.00 | -0.10 | 0.05 | 0.076 |
| rs9847614 | 3 | 126092635 | A | G | 0.13 | 0.99 | -0.20 | 0.08 | 0.011 |
| rs7614708 | 3 | 126092656 | A | G | 0.19 | 1.00 | 0.21 | 0.07 | 2.46E-03 |
| rs7373878 | 3 | 126092928 | G | T | 0.37 | 0.99 | 0.02 | 0.06 | 0.72 |
| rs9864770 | 3 | 126096442 | A | G | 0.13 | 0.90 | -0.19 | 0.08 | 0.021 |
| rs9829173 | 3 | 126101558 | A | T | 0.18 | 1.00 | 0.22 | 0.07 | 1.56E-03 |
| rs4679366 | 3 | 126103881 | C | G | 0.59 | 0.90 | 0.03 | 0.06 | 0.65 |
| rs1532600 | 3 | 126106540 | A | C | 0.95 | 0.94 | 0.10 | 0.12 | 0.43 |
| rs12732 | 3 | 126107258 | A | G | 0.79 | 0.99 | 0.16 | 0.07 | 0.012 |
| rs7430813 | 3 | 126109363 | A | G | 0.79 | 0.99 | 0.16 | 0.07 | 0.012 |
| rs1127233 | 3 | 126109714 | G | T | 0.26 | 0.96 | -0.17 | 0.06 | 6.69E-03 |
| rs9817244 | 3 | 126111749 | A | G | 0.13 | 0.96 | -0.18 | 0.08 | 0.026 |
| rs7621768 | 3 | 126112345 | C | G | 0.44 | 0.97 | 0.00 | 0.06 | 0.96 |
| rs17309490 | 3 | 126112838 | A | C | 0.26 | 0.98 | -0.17 | 0.06 | 5.83E-03 |
| rs2270780 | 3 | 126114817 | A | G | 0.79 | 1.00 | 0.17 | 0.07 | 0.011 |
| rs17309622 | 3 | 126117688 | A | G | 0.19 | 0.99 | 0.21 | 0.07 | 3.05E-03 |
| rs4679165 | 3 | 126118134 | A | G | 0.74 | 0.98 | 0.17 | 0.06 | 5.63E-03 |
| rs4679166 | 3 | 126118202 | C | T | 0.70 | 0.97 | 0.14 | 0.06 | 0.018 |
| rs6778290 | 3 | 126118600 | A | G | 0.15 | 0.84 | 0.25 | 0.08 | 2.37E-03 |
| rs9826731 | 3 | 126118984 | A | G | 0.74 | 0.97 | 0.17 | 0.06 | 5.36E-03 |
| rs9859266 | 3 | 126125525 | C | T | 0.17 | 0.84 | 0.24 | 0.08 | 2.37E-03 |
| rs2877822 | 3 | 126127724 | C | T | 0.78 | 0.98 | 0.10 | 0.07 | 0.13 |
| rs6770196 | 3 | 126128924 | A | C | 0.59 | 0.98 | -0.05 | 0.05 | 0.35 |
| rs4679392 | 3 | 126129284 | A | G | 0.59 | 0.98 | -0.05 | 0.05 | 0.35 |
| rs4679394 | 3 | 126129527 | A | G | 0.15 | 0.98 | -0.16 | 0.07 | 0.036 |
| rs9856999 | 3 | 126129893 | A | G | 0.41 | 0.98 | 0.05 | 0.05 | 0.35 |
| rs6765247 | 3 | 126130417 | G | T | 0.19 | 1.00 | 0.18 | 0.07 | 6.14E-03 |
| rs9824790 | 3 | 126131155 | A | G | 0.78 | 1.00 | 0.09 | 0.06 | 0.17 |
| rs9882808 | 3 | 126131395 | C | T | 0.15 | 1.00 | -0.14 | 0.07 | 0.058 |
| rs1909580 | 3 | 126131510 | A | G | 0.59 | 1.00 | -0.05 | 0.05 | 0.36 |
| rs10804566 | 3 | 126132209 | C | T | 0.11 | 0.40 | 0.29 | 0.14 | 0.037 |
| rs9815561 | 3 | 126137784 | C | T | 0.16 | 0.69 | -0.09 | 0.09 | 0.33 |

# References

1. The Childhood Asthma Management Program (CAMP): design, rationale, and methods. Childhood Asthma Management Program Research Group. Control Clin Trials. 1999 Feb;20(1):91-120.

2. Childhood Asthma Management Program Research Group. Childhood Asthma Man- agement Program Manual for Methacholine Challenge Testing, Version 3.0 (Accession No. PB95-137154) Springfield, VA: National Technical Information Service; 1994.

3. Sutherland ER, Lehman EB, Teodorescu M, Wechsler ME. Body mass index and phenotype in subjects with mild-to-moderate persistent asthma. J Allergy Clin Immunol. 2009 Jun;123(6):1328-34 e1.

4. Szefler SJ, Martin RJ, King TS, Boushey HA, Cherniack RM, Chinchilli VM, et al. Significant variability in response to inhaled corticosteroids for persistent asthma. J Allergy Clin Immunol. 2002 Mar;109(3):410-8.

5. Drazen JM, Israel E, Boushey HA, Chinchilli VM, Fahy JV, Fish JE, et al. Comparison of regularly scheduled with as-needed use of albuterol in mild asthma. Asthma Clinical Research Network. N Engl J Med. 1996 Sep 19;335(12):841-7.

6. Martin RJ, Szefler SJ, Chinchilli VM, Kraft M, Dolovich M, Boushey HA, et al. Systemic effect comparisons of six inhaled corticosteroid preparations. Am J Respir Crit Care Med. 2002 May 15;165(10):1377-83.

7. Boushey HA, Sorkness CA, King TS, Sullivan SD, Fahy JV, Lazarus SC, et al. Daily versus as-needed corticosteroids for mild persistent asthma. N Engl J Med. 2005 Apr 14;352(15):1519-28.

8. Martin RJ, Szefler SJ, King TS, Kraft M, Boushey HA, Chinchilli VM, et al. The Predicting Response to Inhaled Corticosteroid Efficacy (PRICE) trial. J Allergy Clin Immunol. 2007 Jan;119(1):73-80.

9. Lemanske RF, Jr., Allen DB. Choosing a long-term controller medication in childhood asthma. The proverbial two-edged sword. Am J Respir Crit Care Med. 1997 Sep;156(3 Pt 1):685-7.

10. Lemanske RF, Jr., Mauger DT, Sorkness CA, Jackson DJ, Boehmer SJ, Martinez FD, et al. Step-up therapy for children with uncontrolled asthma receiving inhaled corticosteroids. N Engl J Med. 2010 Mar 18;362(11):975-85.

11. Szefler SJ, Phillips BR, Martinez FD, Chinchilli VM, Lemanske RF, Strunk RC, et al. Characterization of within-subject responses to fluticasone and montelukast in childhood asthma. J Allergy Clin Immunol. 2005 Feb;115(2):233-42.

12. Sorkness CA, Lemanske RF, Jr., Mauger DT, Boehmer SJ, Chinchilli VM, Martinez FD, et al. Long-term comparison of 3 controller regimens for mild-moderate persistent childhood asthma: the Pediatric Asthma Controller Trial. J Allergy Clin Immunol. 2007 Jan;119(1):64-72.

13. Strunk RC, Bacharier LB, Phillips BR, Szefler SJ, Zeiger RS, Chinchilli VM, et al. Azithromycin or montelukast as inhaled corticosteroid-sparing agents in moderate-to-severe childhood asthma study. J Allergy Clin Immunol. 2008 Dec;122(6):1138-44 e4.

14. Purcell S, Neale B, Todd-Brown K, Thomas L, Ferreira MA, Bender D, et al. PLINK: a tool set for whole-genome association and population-based linkage analyses. Am J Hum Genet. 2007 Sep;81(3):559-75.

15. Abecasis GR, Cherny SS, Cookson WO, Cardon LR. GRR: graphical representation of relationship errors. Bioinformatics. 2001 Aug;17(8):742-3.
